# Fructose-coated Angstrom silver inhibits osteosarcoma growth and metastasis via promoting ROS-dependent apoptosis through the alteration of glucose metabolism by inhibiting PDK

**DOI:** 10.7150/thno.45858

**Published:** 2020-06-19

**Authors:** Xiong-Ke Hu, Shan-Shan Rao, Yi-Juan Tan, Hao Yin, Ming-Jie Luo, Zhen-Xing Wang, Jin-Hua Zhou, Chun-Gu Hong, Zhong-Wei Luo, Wei Du, Ben Wu, Zi-Qi Yan, Ze-Hui He, Zheng-Zhao Liu, Jia Cao, Yang Wang, Wei-Yi Situ, Hao-Ming Liu, Jie Huang, Yi-Yi Wang, Kun Xia, Yu-Xuan Qian, Yan Zhang, Tao Yue, Yi-Wei Liu, Hong-Qi Zhang, Si-Yuan Tang, Chun-Yuan Chen, Hui Xie

**Affiliations:** 1 Department of Orthopedics, Xiangya Hospital, Central South University, Changsha, Hunan 410008, China.; 2 Movement System Injury and Repair Research Center, Xiangya Hospital, Central South University, Changsha, Hunan 410008, China.; 3 Xiangya Hospital of Central South University - Amcan Pharmaceutical Biotechnology Co. Ltd. Joint Research Center, Changsha, Hunan 410008, China.; 4 Xiangya School of Nursing, Central South University, Changsha, Hunan 410013, China.; 5 Department of Orthopedics, Hunan Provincial People's Hospital, The First Affiliated Hospital of Hunan Normal University, Changsha, Hunan 410005, China.; 6 Department of Rehabilitation, Xiangya Hospital, Central South University, Changsha, Hunan 410008, China.; 7 Department of Sports Medicine, Xiangya Hospital, Central South University, Changsha, Hunan 410008, China.; 8 Institute of Integrative Medicine, Xiangya Hospital, Central South University, Changsha, Hunan 410008, China.; 9 Department of Spine Surgery, Xiangya Hospital, Central South University, Changsha, Hunan 410008, China.; 10 Hunan Key Laboratory of Organ Injury, Aging and Regenerative Medicine, Changsha, Hunan 410008, China.; 11 Hunan Key Laboratory of Bone Joint Degeneration and Injury, Changsha, Hunan 410008, China.; 12 National Clinical Research Center for Geriatric Disorders, Xiangya Hospital, Central South University, Changsha, Hunan 410008, China.; 13 Key Laboratory of Biological Nanotechnology of National Health Commission, Changsha, Hunan 410008, China.

**Keywords:** Ångstrom-scale silver particles, osteosarcoma, reactive oxygen species, glucose metabolism, pyruvate dehydrogenase kinase

## Abstract

Osteosarcoma is a common malignant bone cancer easily to metastasize. Much safer and more efficient strategies are still needed to suppress osteosarcoma growth and lung metastasis. We recently presented a pure physical method to fabricate Ångstrom-scale silver particles (AgÅPs) and determined the anti-tumor efficacy of fructose-coated AgÅPs (F-AgÅPs) against lung and pancreatic cancer. Our study utilized an optimized method to obtain smaller F-AgÅPs and aimed to assess whether F-AgÅPs can be used as an efficient and safe agent for osteosarcoma therapy. We also investigated whether the induction of apoptosis by altering glucose metabolic phenotype contributes to the F-AgÅPs-induced anti-osteosarcoma effects.

**Methods:** A modified method was developed to prepare smaller F-AgÅPs. The anti-tumor, anti-metastatic and pro-survival efficacy of F-AgÅPs and their toxicities on healthy tissues were compared with that of cisplatin (a first-line chemotherapeutic drug for osteosarcoma therapy) in subcutaneous or orthotopic osteosarcoma-bearing nude mice. The pharmacokinetics, biodistribution and excretion of F-AgÅPs were evaluated by testing the levels of silver in serum, tissues, urine and feces of mice. A series of assays *in vitro* were conducted to assess whether the induction of apoptosis mediates the killing effects of F-AgÅPs on osteosarcoma cells and whether the alteration of glucose metabolic phenotype contributes to F-AgÅPs-induced apoptosis.

**Results:** The newly obtained F-AgÅPs (9.38 ± 4.11 nm) had good stability in different biological media or aqueous solutions and were more effective than cisplatin in inhibiting tumor growth, improving survival, attenuating osteolysis and preventing lung metastasis in osteosarcoma-bearing nude mice after intravenous injection, but were well tolerated in normal tissues. One week after injection, about 68% of F-AgÅPs were excreted through feces. F-AgÅPs induced reactive oxygen species (ROS)-dependent apoptosis of osteosarcoma cells but not normal cells, owing to their ability to selectively shift glucose metabolism of osteosarcoma cells from glycolysis to mitochondrial oxidation by inhibiting pyruvate dehydrogenase kinase (PDK).

**Conclusion:** Our study suggests the promising prospect of F-AgÅPs as a powerful selective anticancer agent for osteosarcoma therapy.

## Introduction

Osteosarcoma is a common malignant bone tumor in children and young adults with a high rate of recurrence and a strong tendency to metastasize to distant organs such as the lungs 1-3. Current treatment options for osteosarcoma mainly include neoadjuvant chemotherapy, tumor amputation and postoperative chemotherapy, which enhance the survival rate of patients with osteosarcoma 1-3. Nevertheless, death from respiratory failure due to lung metastases remains a significant problem 1. Moreover, the use of chemotherapeutic agents (cisplatin, doxorubicin, methotrexate, *etc.*) is often limited by their severe side effects and resistance of cancer cells to these drugs 2, 4. Thus, it is essential to develop much safer and more efficient strategies to suppress osteosarcoma growth and lung metastasis.

Nanoparticles (NPs) have emerged as a new promising strategy for cancer therapy 5. The unique properties of NPs, such as their ultra-small size and large surface area to volume ratio, make them desirable as therapeutic agents 6, 7. NPs in the range of 5.5-100 nm are apt to accumulate in the tumor sites through the leaky blood vessels of tumors, but can be restricted from normal vasculature (that needs sizes > 2 nm) and avoid the first-pass elimination by the kidney (which requires diameters > 5.5 nm) 8-11. This phenomenon is known as enhanced permeability and retention (EPR) effect, which enhances the bioavailability of NPs and reduces their systemic toxicity 10-13. NPs with smaller size are more easily to penetrate into tumors and evoke stronger anti-cancer activities 14-16. Hence, it becomes important to fabricate NPs possessing the smallest possible size, high tumor-targeting efficiency and favorable pharmacokinetics for tumor therapy 16.

Silver has been employed for biomedical purposes since ancient time owing to its anti-microbial properties 17, 18. Recent researches on Dalton's lymphoma ascites tumor model 19, glioblastoma- or melanoma-bearing mice 20, 21, and lung or breast cancer-bearing mice 22-24 have demonstrated the efficacy of silver NPs (AgNPs) in suppressing tumor growth. More recently, we reported a pure physical method, which avoided the use of any hazardous biological and chemical reducing agents, to fabricate ultra-small silver particles reaching the Ångstrom (Ång; one-tenth of nanometer) sizes 16. We used fructose to stabilize and disperse these Ång-scale silver particles (AgÅPs) and confirmed that the fructose-coated AgÅPs (F-AgÅPs; 22.8 ± 5.4 nm) could induce anti-tumor effects in lung and pancreatic cancer-bearing nude mice without inducing obvious systemic toxicity 16. These findings prompted us to explore whether F-AgÅPs can be used as an efficient and safe agent for osteosarcoma therapy.

Here, we optimized the coating conditions to obtain smaller F-AgÅPs (9.38 ± 4.11 nm) and assessed the *in vitro* toxicities of F-AgÅPs against osteosarcoma cell lines and primary osteosarcoma cells from patients. *In vivo*, we compared the anti-tumor and anti-metastatic efficacy and the safety between F-AgÅPs and the first-line chemotherapeutic drug cisplatin in osteosarcoma-bearing nude mice. We determined whether F-AgÅPs can prolongate the survival time of these mice and evaluated the pharmacokinetics, biodistribution and excretion of F-AgÅPs. As we found that F-AgÅPs selectively caused mitochondrial membrane potential (Δ*Ψ*_m_) reduction and reactive oxygen species (ROS)-dependent apoptotic death in osteosarcoma cells rather than in normal cells, and did not induce any notable toxicity in normal tissues after intravenous administration, we further investigated the underlying mechanism.

The unique metabolic profile (aerobic glycolysis) and high Δ*Ψ*_m_ of cancer cells confer their resistance to apoptosis 25. Switching of metabolism from glycolysis to glucose oxidation in cancer cells increases ROS generation and decreases Δ*Ψ*_m_ (mitochondrial depolarization), thus triggering mitochondria-dependent apoptosis 25. Pyruvate dehydrogenase kinase (PDK) is a mitochondrial enzyme that induces metabolic switch from glucose oxidation to glycolysis in cancer cells by selectively deactivating pyruvate dehydrogenase (PDH) through the phosphorylation of the E1α subunit 25, 26. Thus, we focused on energy metabolism to explore whether the alteration of metabolic phenotype of osteosarcoma cells by modulating PDK/PDH contributes to the selective anti-tumor efficacy of F-AgÅPs.

## Results

### Characterization of F-AgÅPs

**Figure 1:**
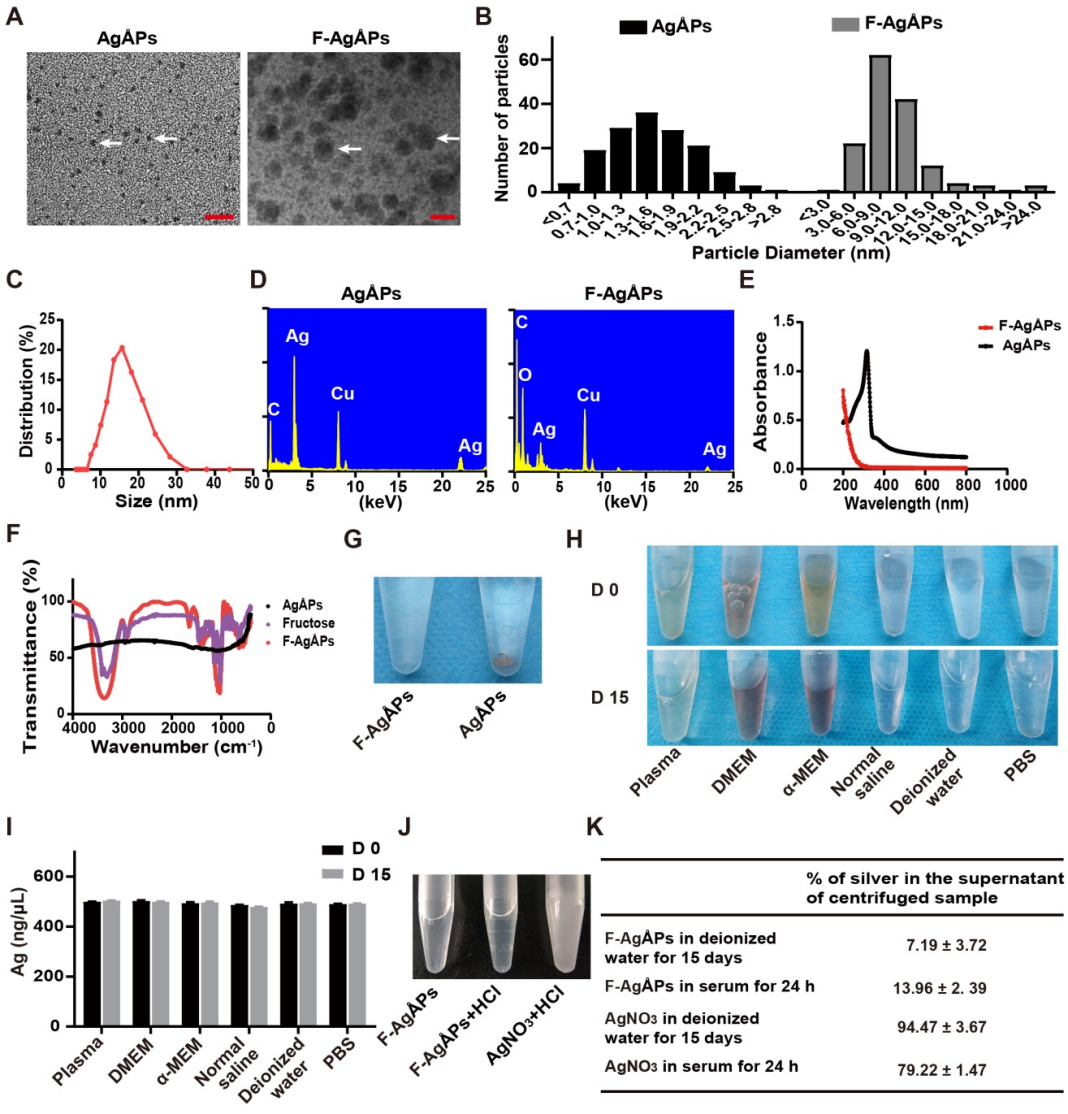
** Characterization of F-AgÅPs.** (**A**) Morphologies of AgÅPs and F-AgÅPs under a transmission electron microscope. Scale bar: 10 nm. (**B**) Size distribution of AgÅPs (15.53 ± 5.1 Ång; *n* = 150) and F-AgÅPs (9.38 ± 4.11 nm; *n* = 150) under the transmission electron microscope. (**C**) Hydrodynamic diameter distribution of F-AgÅPs measured by DLS. (**D**) Elemental constitution of AgÅPs and F-AgÅPs analyzed by EDS. (**E**) UV-Vis-NIR absorption spectra of AgÅPs (black line) and F-AgÅPs (red line). (**F**) FT-IR absorption spectra of fructose (purple line), AgÅPs (black line) and F-AgÅPs (red line). (**G**) Photographs of AgÅPs and F-AgÅPs aqueous solutions left for one month at room temperature. (**H** and** I**) Photographs of F-AgÅPs in plasma, cell culture media (including DMEM and α-MEM), normal saline, deionized water and PBS left at room temperature for 15 days (**H**) and silver concentration in their supernatant measured by ICP-MS (**I**). *n* = 3 *per* group. (**J**) Photographs of F-AgÅPs and AgNO_3_ suspensions after being mixed with HCl. (**K**) The percentages of silver in the supernatant of the centrifuged F-AgÅPs and AgNO_3_ preparations in deionized water for 15 days and in serum for 24 h. *n* = 4 *per* group. Data are shown as mean ± SD. ^*^P < 0.01, ^**^P < 0.01, ^***^P < 0.001.

AgÅPs were prepared by an enclosed, automatic and high-efficiency evaporation-condensation system as previously described in detail 16. As shown in **Figure 1**, **A** and **B**, the naked AgÅPs showed sphere-shaped morphologies under a transmission electron microscope, with diameters ranging from 1 to 30 Ång (15.53 ± 5.10 Ång). An optimized method by changing the power and frequency of ultrasonic dispersion system was adopted to prepare smaller F-AgÅPs, in order to simultaneously take into account the dispersion, stability, tumor-targeting and anti-tumor efficiency, and favorable pharmacokinetics of AgÅPs. As compared to our previously reported F-AgÅPs (22.8 ± 5.4 nm), the obtained F-AgÅPs in this study displayed much smaller physical sizes and hydrodynamic diameters, as revealed by the image and size quantification of F-AgÅPs under the transmission electron microscope (9.38 ± 4.11 nm; **Figure 1**, **A** and **B**) and by the result of dynamic light scattering (DLS) analysis (15.75 ± 6.94 nm; **Figure 1C**), respectively. This improvement will make them much more suitable for cancer therapy. The elemental constitution of AgÅPs and F-AgÅPs was analyzed by energy dispersive spectroscopy (EDS). High level of elemental silver signal was detected in AgÅPs (**Figure 1D**). The presence of elemental copper and carbon in AgÅPs was most likely attributed to the inevitable element contamination during sample preparation, which required the use of carbon fiber membrane and copper grid. The decreased proportion of silver and enhanced proportion of carbon and oxygen in F-AgÅPs suggested that fructose is successfully coated on AgÅPs (**Figure 1D**). The ultraviolet-visible near-infrared (UV-Vis-NIR) absorption spectra of AgÅPs and F-AgÅPs were illustrated in **Figure 1E**. The localized surface plasmon resonance (LSPR) peak wavelength (λ max) of AgÅPs was centered around 316 nm, which showed a blue shift compared with the previously reported AgNPs (>400 nm) 14, 23, 24, a phenomenon likely due to the much smaller size of AgÅPs than AgNPs 27. The LSPR peak of F-AgÅPs disappeared, suggesting that AgÅPs are successfully embedded into fructose. The infrared transmittance spectra of F-AgÅPs were tested by Fourier-transform infrared (FT-IR) spectrometer. As shown in **Figure 1F**, both fructose and F-AgÅPs, but not the naked AgÅPs, exhibited a narrow band (centered at 1025.55 cm^-1^ and 1032.35 cm^-1^, respectively) due to CO stretching vibration, and a strong and broad OH stretching band (centered at 3312.70 cm^-1^ and 3361.87 cm^-1^, respectively), suggesting that fructose is coated on AgÅPs and the package of AgÅPs induces slight changes in FT-IR absorption peaks of fructose. **Figure 1G** shows that AgÅPs aggregated in deionized water when the samples were left for one month at room temperature, whereas F-AgÅPs suspension remained transparent, indicating that encapsulation within fructose makes AgÅPs stable in aqueous solution. Consistent with that observed in deionized water, photographs of F-AgÅPs in plasma, cell culture media (including DMEM and α-MEM), normal saline and PBS showed that no aggregates were formed after these F-AgÅPs suspensions were left at room temperature for 15 days (**Figure 1H**). Inductively coupled plasma mass spectrometry (ICP-MS) showed comparable levels of silver in the supernatant of these F-AgÅPs-containing biological media or aqueous solutions at days 15 and day 0, which further confirmed the good stability and dispersion of F-AgÅPs in different biological media/aqueous solutions (**Figure 1I**).

Since silver particles can release silver ions *in vitro* and *in vivo* 28, we assessed whether F-AgÅPs can release silver ions in deionized water and in serum isolated from mice. As silver ions can react with Cl^-^ to from white precipitation AgCl, we mixed excessive HCl with F-AgÅPs that were dissolved in deionized water and left at room temperature for 15 days. The same silver concentration of AgNO_3_ was served as the positive control to react with HCl. As shown in **Figure 1J**, the transparent solution of AgNO_3_ became turbid after being mixed with HCl, but F-AgÅPs suspension remained clear with the addition of HCl, suggesting that there are no enough silver ions released from F-AgÅPs to react with HCl to form AgCl precipitates. F-AgÅPs and AgNO_3_ preparations in deionized water for 15 days and in serum for 24 h were centrifuged and the amounts of silver in the supernatant were tested by ICP-MS. The result showed that only a small amount of silver was found in the supernatant of F-AgÅPs preparation in deionized water (7.19% ± 3.72%) and in serum (13.96% ± 2. 39%), whereas 94.47% ± 3.67% and 79.22% ± 1.47% of silver, respectively, was detected in the supernatant of AgNO_3_ preparation in deionized water and in serum (**Figure 1K**). These findings suggest that F-AgÅPs are able to release silver ions, but Ag particles are likely the major mediator of the function of F-AgÅPs.

### Anti-tumor activities of F-AgÅPs* in vitro*

**Figure 2:**
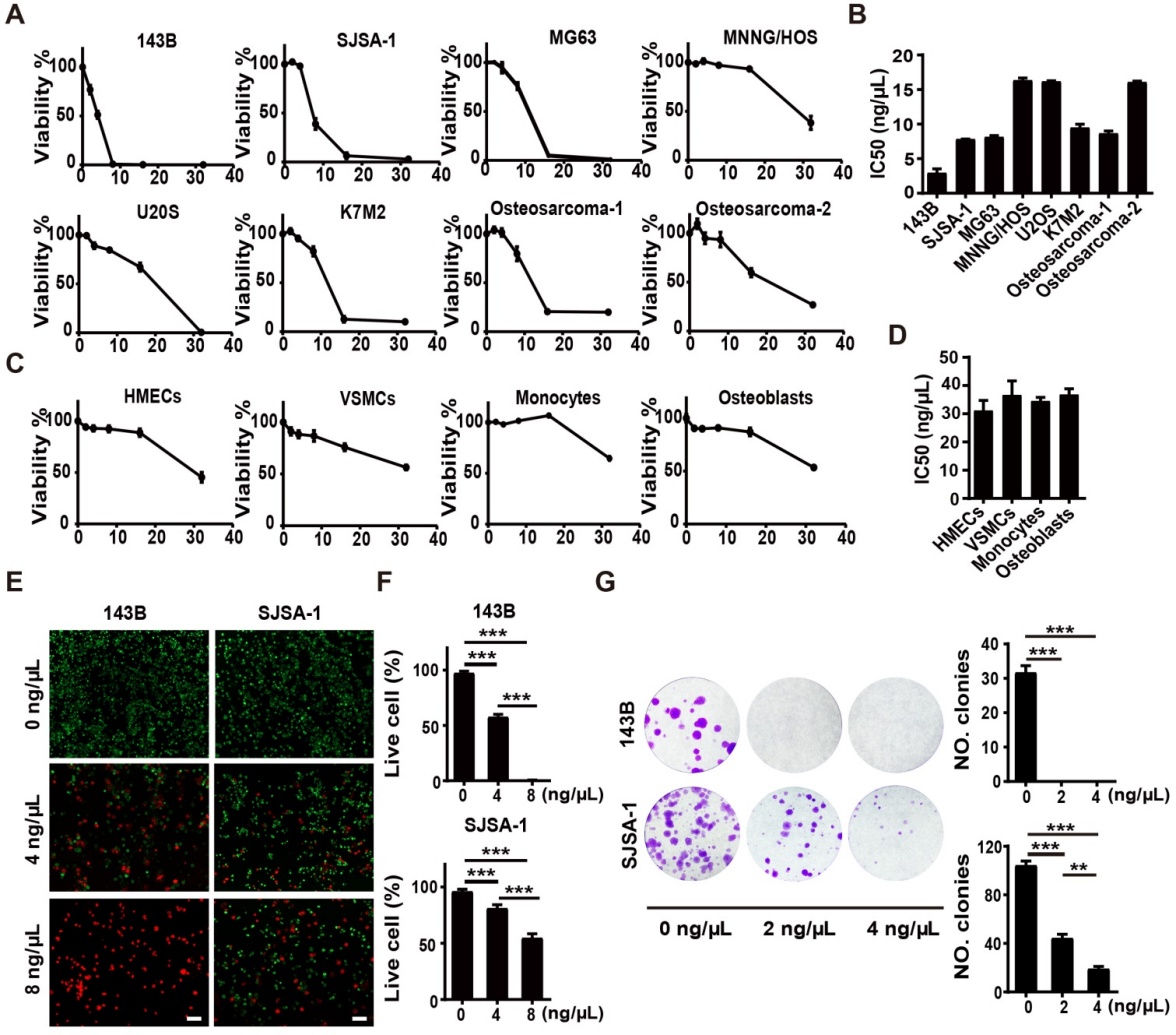
** Anti-tumor activities of F-AgÅPs* in vitro*.** (**A**) CCK-8 analysis of the viability of human or mouse osteosarcoma cell lines and two patients-derived primary osteosarcoma cells receiving different treatments for 24 h. *n* = 5 *per* group. (**B**) IC50 values of F-AgÅPs for osteosarcoma cells in (**A**). *n* = 3 *per* group. (**C**) CCK-8 analysis of the viability of human normal cell lines HMECs and VSMCs as well as mouse primary monocytes and osteoblasts. *n* = 5 *per* group. (**D**) IC50 values of F-AgÅPs for normal cells in (**C**). *n* = 3 *per* group. (**E**) Representative images of calcein-AM/PI staining of 143B and SJSA-1 receiving different treatments for 24 h. Scale bar: 100 µm. (**F**) Quantification of the percentages of live cells (calcein-AM^+^PI^-^) in (**E**). *n* = 3 *per* group. (**G**) Representative images and quantification of the crystal violet-stained colonies formed by 143B and SJSA-1 receiving different treatments for 14 days. *n* = 3 *per* group. Data are shown as mean ± SD.^*^P < 0.01, ^**^P < 0.01, ^***^P < 0.001.

To explore the toxicities of F-AgÅPs against osteosarcoma cells, we firstly tested the effects of F-AgÅPs on the viability of a class of murine or human osteosarcoma cell lines. Cell counting kit-8 (CCK-8) assays showed that incubation with F-AgÅPs for 24 h inhibited the viability of osteosarcoma cells in a dose-dependent manner (**Figure 2A**). The values of the half-maximal inhibitory concentration (IC50) revealed that 143B (IC50 = 2.92 ± 1.06 ng/μL) exhibited the highest sensitivity to F-AgÅPs among these osteosarcoma cell lines, followed by SJSA-1 (IC50 = 7.81 ± 0.05 ng/μL) (**Figure 2B**), either of which has a high malignancy and high tendency to metastasize 29, 30. The IC50 values of F-AgÅPs against MNNG-HOS and U2OS, respectively, were 16.31 ± 0.61 ng/μL and 16.15 ± 0.23 ng/μL (**Figure 2B**), indicating that they are relatively less sensitive to F-AgÅPs. We also isolated tumor cells from two patients with advanced osteosarcoma and analyzed their sensitivity to F-AgÅPs. CCK-8 assay demonstrated that F-AgÅPs were effective in inhibiting the survival of these primary osteosarcoma cells with IC50 values of 8.65 ± 0.63 ng/μL and 16.06 ± 0.27 ng/μL, respectively (**Figure 2A** and **B**). However, the toxicity of F-AgÅPs was relatively lower to normal cell lines or primary cells including human microvascular endothelial cells (HMECs), vascular smooth muscle cells (VSMCs) as well as mouse monocytes and osteoblasts (**Figure 2C** and **D**), suggesting a promising potential of F-AgÅPs as a safe agent for osteosarcoma therapy. Calcein-AM/propidium iodide (PI) staining was also conducted to assess cell viability. As shown in **Figure 2E** and **F**, F-AgÅPs caused a marked reduction of the percentages of living 143B and SJSA-1 (calcein-AM^+^PI^-^) and the effect was enhanced with the increase of the dose of F-AgÅPs.

We then assayed the influence of F-AgÅPs on colony formation (a parameter positively correlated with increased cancer cell malignancy 31) of 143B and SJSA-1. As shown in **Figure 2G**, 2 ng/μL F-AgÅPs were sufficient to significantly repress their ability to form colonies, especially 143B, which could not form colonies after exposure to F-AgÅPs. With the increase of concentration, the inhibitory effect of F-AgÅPs on colony formation of SJSA-1 was enhanced (**Figure 2G**). Thus, F-AgÅPs can suppress the malignancy of osteosarcoma cells.

### F-AgÅPs inhibit the growth and lung metastasis of osteosarcoma

**Figure 3:**
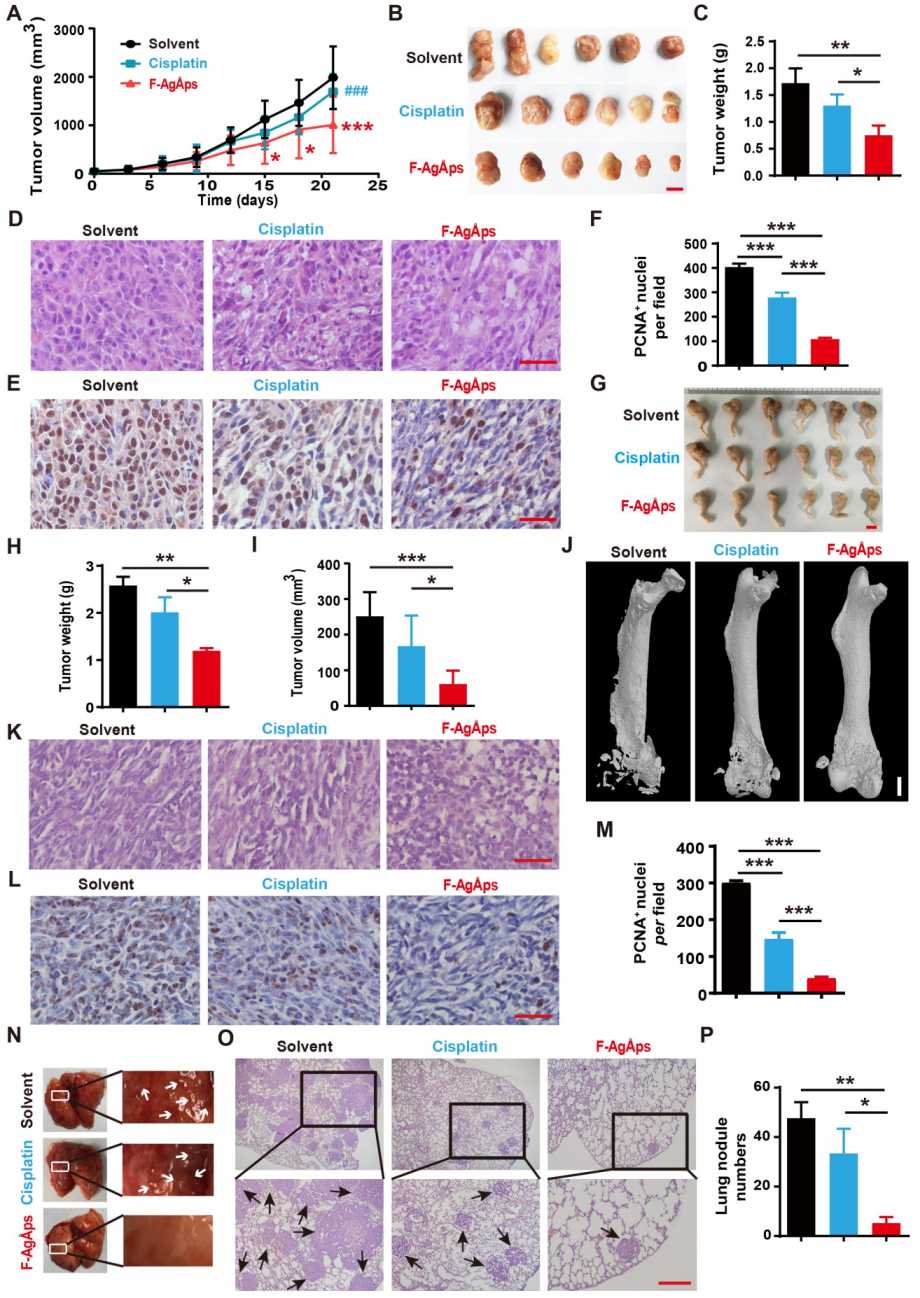
** F-AgÅPs inhibit the growth and lung metastasis of osteosarcoma.** (**A**) Tumor volumes of subcutaneous 143B xenograft-bearing mice in different treatment groups during an observation period of 21 days. *n* = 6 *per* group. (**B** and** C**) Photographs (**B**) and weights (**C**) of tumor samples from mice in (**A**) at days 21. Scale bar: 1 cm. *n* = 6 *per* group. (**D**) Representative images of the H&E-stained tumor sections from samples in (**B**). Scale bar: 50 µm. (**E** and** F**) Representative PCNA staining images (**E**) and quantification of the PCNA-positive cell numbers (**F**) in tumor sections from samples in (**B**). Scale bar: 50 µm. *n* = 3 *per* group. (**G**) Photographs of the right hindlimb samples from orthotopic SJSA-1-bearing mice receiving different treatments for 21 days. Scale bar: 1 cm. (**H** and** I**) Tumor weights (**H**) and volumes (**I**) of samples in (**G**). *n* = 6 *per* group. (**J**) Representative µCT images of the mouse right hindlimb specimens in (**G**). Scale bar: 1 mm. (**K** and **L**) Representative H&E (**K**) and PCNA (**L**) staining images of tumor specimens in (**G**). Scale bar: 50 µm. (**M**) Quantification of the PCNA-positive cell numbers. *n* = 3 *per* group. (**N**) Gross view of lungs from mice in (**G**). (**O** and** P**) Representative images of the H&E-stained lung sections (**O**) and quantification of the metastatic tumor nodule numbers (**P**). Scale bar: 200 µm. *n* = 3 *per* group. Data are shown as mean ± SD. ^*#^*P* < 0.05, ^**/##^*P* < 0.01, ^***/###^*P* < 0.001. For (**A**):^ *^P < 0.05 vs. solvent group, **^#^***P* < 0.05 vs. F-AgÅPs group.

We then generated subcutaneous 143B xenografts in nude mice, and compared the anti-tumor efficiency of F-AgÅPs and cisplatin (a first-line chemotherapeutic drug for osteosarcoma therapy) 32 against osteosarcoma *in vivo*. **Figure 3A** shows the tumor sizes of 143B xenograft-bearing nude mice during an observation period of 21 days after intravenous injection of F-AgÅPs, cisplatin or solvent (normal saline). Both F-AgÅPs and cisplatin suppressed the growth of 143B tumor xenografts, but F-AgÅPs exhibited a much higher tumor inhibition ratio (49.19%) relative to cisplatin (14.53%) on day 21 (**Figure 3A**). The higher anti-tumor efficiency of F-AgÅPs than cisplatin was determined by gross observation of tumor specimens and their weights at the termination of the experiment (**Figure 3B** and **C**). Hematoxylin and eosin (H&E) staining of tumor sections showed the presence of hypercellularity and hyperchromasia in the solvent-treated control mice (**Figure 3D**). After treatment with F-AgÅPs or cisplatin, many cells in tumor tissues became necrotic, vacuolated or shrunken with fragmented or pyknotic nuclei, and the changes were much more evident in the F-AgÅPs-treated mice (**Figure 3D**). Immunohistochemical staining for proliferating cell nuclear antigen (PCNA) showed that both F-AgÅPs and cisplatin markedly repressed cell proliferation in the tumor sites, but the inhibitory effects of F-AgÅPs were much higher than that of cisplatin (**Figure 3E** and **F**). No evidence of lung metastasis was detected in these mice, as indicated by gross view of lungs and H&E staining images of lung sections (**Figure S1A**).

We also established orthotopic models of osteosarcoma in nude mice by injection of SJSA-1 cells into bone marrow space of the right femurs, and evaluated the effects of F-AgÅPs and cisplatin on tumor progression after injection weekly for 3 weeks. As revealed by gross observation of the right hindlimbs (**Figure 3G**), tumor weights (**Figure 3H**) and tumor volumes (**Figure 3I**), F-AgÅPs caused much higher levels of tumor growth inhibition than cisplatin. Microcomputed tomography (μCT) scanning was conducted to evaluate bone structure changes. As shown in **Figure 3J**, serious lytic bone destruction ("moth-eaten" appearance) and cortical bone loss occurred in the distal femurs of the vehicle-treated mice. Injection of F-AgÅPs and cisplatin profoundly prevented bone damage and most bone tissues were maintained in these groups, especially in those treated with F-AgÅPs (**Figure 3J**). H&E staining revealed large areas of necrosis in the F-AgÅPs-treated mice, but the changes were much weaker in the cisplatin-treated mice (**Figure 3K**). PCNA staining demonstrated a stronger ability of F-AgÅPs to suppress cell proliferation in the tumor sites compared with cisplatin (**Figure 3**, **L** and **M**). Gross appearance of lung and H&E staining for lung sections showed large numbers of metastatic nodules in the solvent-treated mice (**Figure 3N-P**). Injection of F-AgÅPs and cisplatin significantly reduced the numbers of pulmonary tumor nodules, but the anti-metastatic effect of F-AgÅPs was stronger than cisplatin (**Figure 3N-P**). These results suggest that F-AgÅPs are more competent than cisplatin to inhibit osteosarcoma growth and protect against osteosarcoma-induced osteolysis and lung metastasis.

### F-AgÅPs enhance survival without inducing obvious toxicity in osteosarcoma- bearing mice

**Figure 4:**
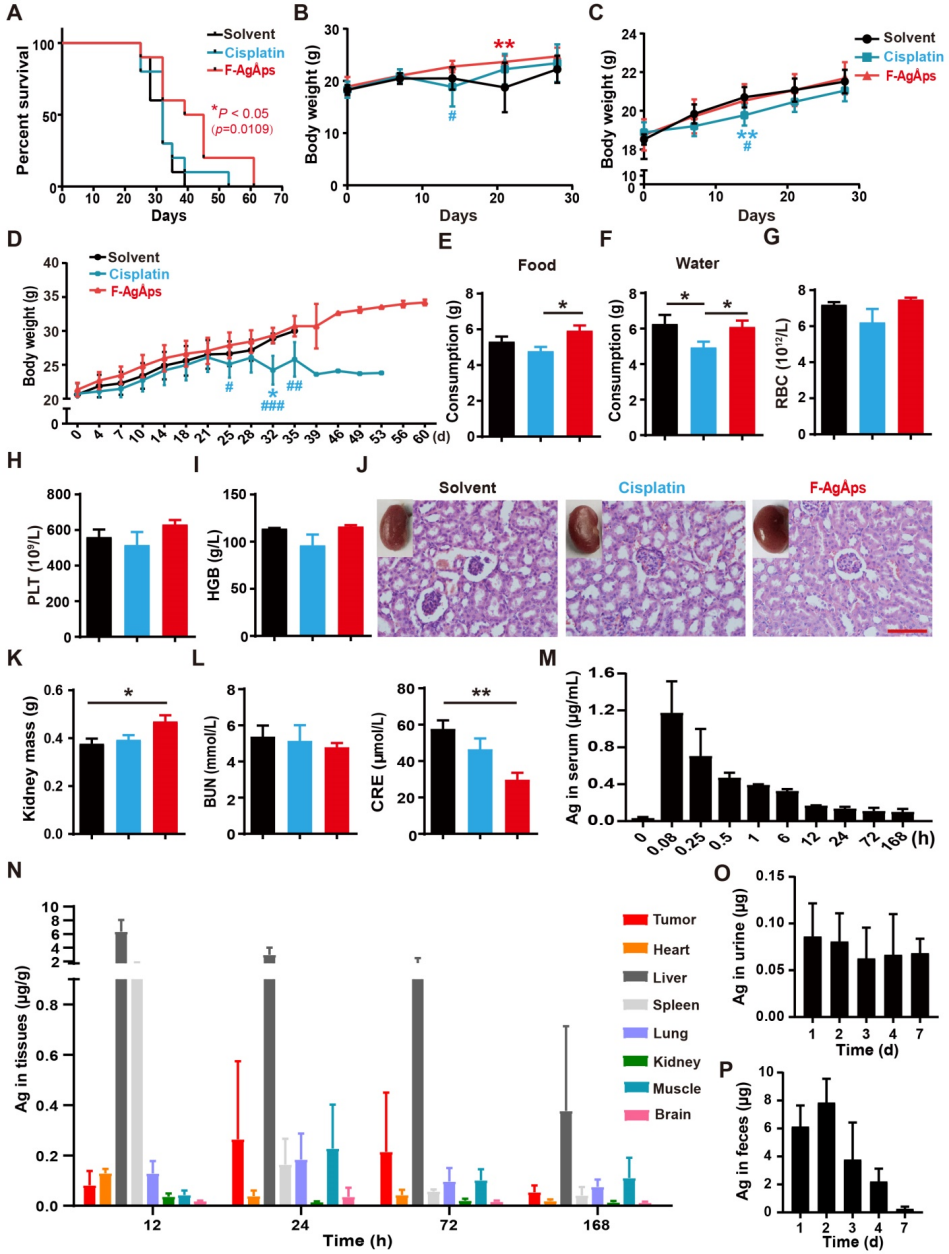
** Toxicities, pharmacokinetics, biodistribution and excretion of F-AgÅPs in osteosarcoma-bearing mice.** (**A**) Kaplan-Meier survival curves of subcutaneous 143B-bearing mice. *n* = 10 *per* group. (**B** and** C**) Body weights of subcutaneous 143B (**B**) or orthotopic SJSA-1 (**C**) xenografts-bearing mice during the assessment of anti-tumor efficacy of F-AgÅPs. *n* = 6 *per* group. (**D**) Body weights of mice in (**A**) were record twice a week until all mice died. (**E** and **F**) Food and water consumption of mice in (**A**) were monitored from day 21 to 28. Average daily food (**E**) and water (**F**) intakes were shown. *n* = 8-10 *per* group. (**G-I**) The numbers/levels of RBC (**G**), PLT (**H**) and HGB (**I**) in blood samples from mice in (**B**) at days 21. *n* = 5 *per* group. (**J**) Gross view and H&E staining images of kidneys from mice in (**B**) at days 21. Scale bar: 100 µm*.* (**K**) The weights of kidney. *n* = 6 *per* group. (**L**) The serum levels of BUN and CRE. *n* = 5 *per* group. (**M**) Blood concentration-time curve of silver in orthotopic SJSA-1-bearing mice untreated (0 min) or treated with F-AgÅPs for the indicated times. *n* = 5 *per* time point. (**N**) Tissue distribution of silver after F-AgÅPs treatment for the indicated times. *n* = 5 *per* time point. (**O** and **P**) Daily excretion levels of silver through urine (**O**) and feces (**P**). *n* = 5. Data are shown as mean ± SD.^*#^*P* < 0.05, ^**/##^*P* < 0.01, ^***/###^*P* < 0.001. For (**B-D**):^ *^P < 0.05 vs. solvent group, **^#^***P* < 0.05 vs. F-AgÅPs group.

We then compared the long-term survival of subcutaneous 143B-bearing nude mice. As indicated by the Kaplan-Meier survival curves, treatment with F-AgÅPs, but not cisplatin, induced a statistically significant increase in the survival rate of 143B-bearing mice compared with solvent group (**Figure 4A**).

When assessing the effects of F-AgÅPs on tumor growth in subcutaneous 143B-bearing mice, body weight loss was observed in mice treated with solvent or cisplatin for 14 days, but not in those treated with F-AgÅPs (**Figure 4B**). In orthotopic SJSA-1-bearing mice, continued increases of body weights occurred in all treatment groups, but the cisplatin-treated mice had the lowest body weights compared to other groups (**Figure 4C**). In the experiment for comparing the effects of F-AgÅPs and cisplatin on survival, the cisplatin-treated mice also had the lowest body weights among all groups (**Figure 4D**). Their body weights began to decrease after treatment for 25 days and statistically significant reductions were observed at days 32 relative to control group and at days 25, 32 and 35 compared with F-AgÅPs group (**Figure 4D**). Food and water intake were monitored from day 21 to day 28. As shown in **Figure 4E** and **F**, the daily consumption of food and water was markedly decreased in the cisplatin-treated mice compared to the solvent- or/and F-AgÅPs-treated mice (**Figure 4E** and **F**). These data, along with the inhibitory effects of cisplatin on tumor burden, suggest that cisplatin treatment may induce side effects. The mice treated with F-AgÅPs had similar or higher body weights and daily food/water intake compared to the control mice (**Figure 4B-F**), suggesting a good tolerability of F-AgÅPs at the therapeutically effective dose.

Myelosuppression often occurs in cisplatin-based chemotherapy 33, 34. Consistently, routine blood test showed that cisplatin treatment for 21 days induced decreases in the levels of red blood cells (RBC), platelets (PLT) and hemoglobin (HGB) in mice bearing subcutaneous 143B, but only by trend (**Figure 4G-I** and **Table S1**). No signs of myelosuppression were detected in mice treated with F-AgÅPs (**Figure 4G-I** and **Table S1**). Tissue toxicities of F-AgÅPs and cisplatin were assessed in subcutaneous 143B xenografts-bearing mice. Cisplatin treatment usually induces dose-dependent tubular necrosis 35. H&E staining showed the presence of mild dilated renal tubules in the cisplatin-treated mice, but no obvious tubular necrosis was found in these mice (**Figure 4J**). Surprisingly, some of the solvent-treated mice showed atrophic glomeruli with hyperchromatic nuclei, whereas the mice in other groups did not show this change (**Figure 4J**), which might be due to the invasion of osteosarcoma cells to kidney in the control mice. The lower levels of kidney weights and higher levels of serum creatinine (CRE) in the solvent-treated control mice than other groups, especially than the mice treated with F-AgÅPs (**Figure 4K** and **L**), further indicated that renal injury might occur in the control mice. No renal pathologic alterations, kidney weight loss and increases of the serum levels of blood urea nitrogen (BUN) and CRE were found in the F-AgÅPs-treated mice (**Figure 4J-L**). All mice did not show significant changes in weights, gross appearance and histological structures of lung, heart, liver, spleen and brain (**Figure S1A-E**). These results further suggest the minimal toxicity of F-AgÅPs superior to cisplatin in osteosarcoma-bearing mice.

### Serum kinetics, tissue distribution and excretion of F-AgÅPs in osteosarcoma- bearing mice

The pharmacokinetics, tissue distribution and excretion of F-AgÅPs (1.5 mg/kg) were assessed in orthotopic SJSA-1-bearing mice. As revealed by ICP-MS data, the concentration of silver in serum at 5 min after injection was the highest (1.172 ± 0.34 μg/mL) among all time points (**Figure 4M**). A rapid decline in the serum level of silver (0.705 ± 0.29 μg/mL) occurred at 15 min after treatment (**Figure 4M**). At 12 h after injection, the serum level of silver was low (0.167 ± 0.004 μg/mL) and decreased very slowly (**Figure 4M**). The serum concentration-time data for F-AgÅPs conformed to the two-compartment model with the distribution half-life (t_1/2α_) of 0.22 ± 0.09 h and the elimination half-life (t_1/2β_) of 32.25 ± 5.44 h. Consistent with the biodistribution feature in pancreatic cancer mouse models 16, F-AgÅPs efficiently accumulated into tumor tissues, where the silver levels were higher than that in heart, spleen, kidney, and brain after injection of F-AgÅPs for 24 h, 3 days and 7 days (**Figure 4N**). Very high levels of silver were detected in the liver and spleen at 12 h after injection, in accordance with previous evidence that NPs easily accumulate in liver and spleen due to the uptake by the mononuclear phagocyte system (MPS) 11, 36, 37. Excretion levels of silver *via* urine and feces were shown in **Figure 4**, **O** and **P**. Silver excretion by urine reached to its peak (0.093 ± 0.034 μg/d) in the first 24 h after injection and decreased to 0.066 ± 0.035 μg/d at day 3 after treatment. The total amount of silver excreted by urine for 7 days was 0.392 ± 0.06 μg, which was less than 2% of total body burden of F-AgÅPs (30 μg for a nude mouse weighing about 20 g). In the entire experimental period, silver excretion by urine was much lower than that by feces, suggesting the elimination of F-AgÅPs by biliary excretion. The highest level (7.853 ± 1.702 μg/d) of silver excretion through feces was detected at day 2 after F-AgÅPs injection. Subsequently, the excreted silver *via* feces was constantly decreased. The cumulative amount of silver excreted through feces for 7 days was about 20.238 ± 2.947 μg, which was close to 68% of the total body burden of F-AgÅPs. Thus, about 30% of F-AgÅPs remained in the body at day 7 after injection.

### ROS-dependent apoptosis mediates F-AgÅPs-induced osteosarcoma cell death

**Figure 5:**
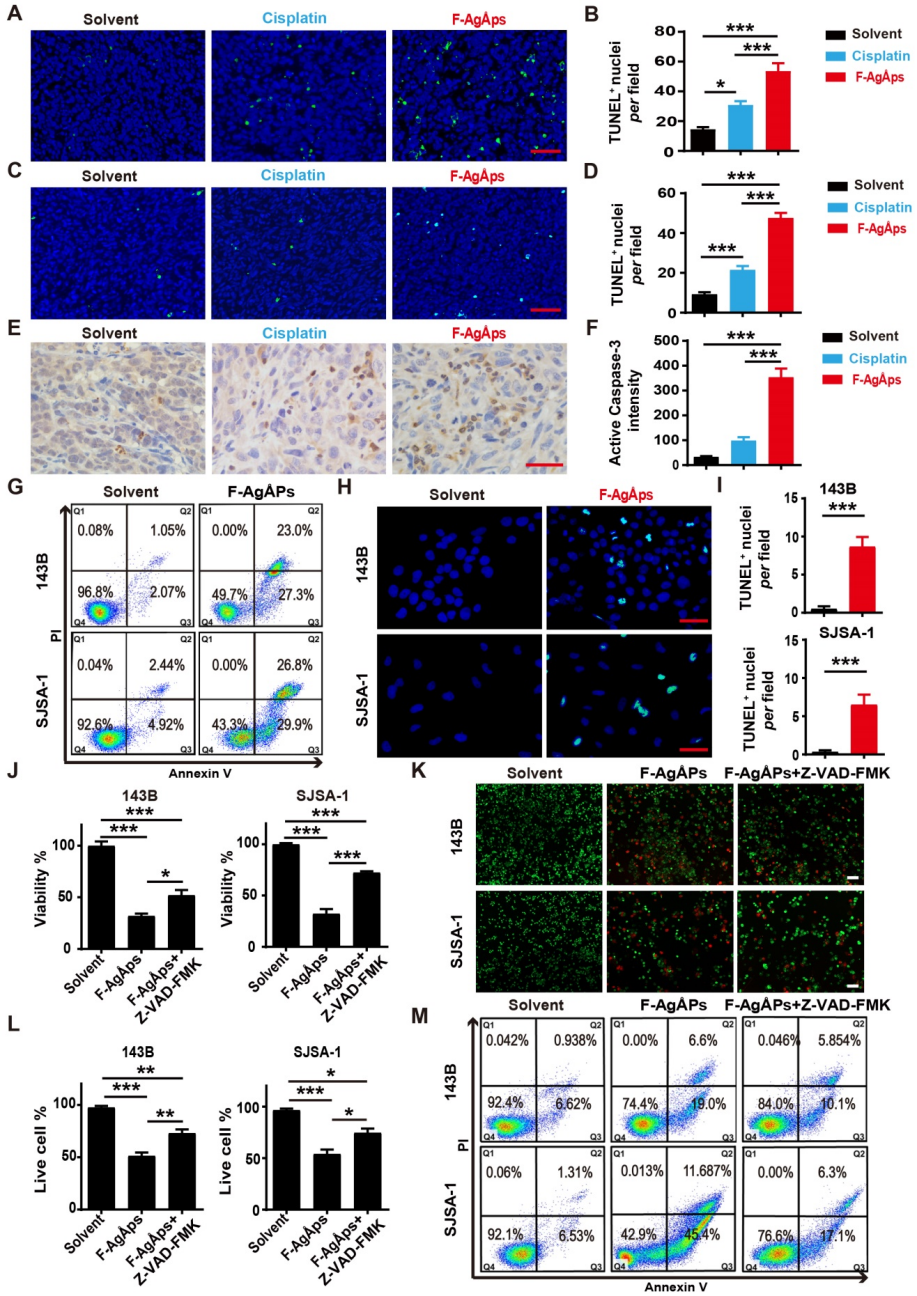
** F-AgÅPs induce osteosarcoma cell death through the promotion of apoptosis.** (**A-D**) Representative images of TUNEL staining and quantification of the apoptotic cell numbers in tumor sections from subcutaneous 143B (**A** and** B**) or orthotopic SJSA-1 (**C** and** D**) xenografts-bearing mice treated with solvent, F-AgÅPs or cisplatin for 21 days. Scale bar: 50 µm. *n* = 3 *per* group. (**E** and **F**) Representative active caspase-3 staining images (**E**) and quantification of the mean staining intensity (**F**) in tumor sections from mice in (**A** and** B**). Scale bar: 50 µm. *n* = 3 *per* group. (**G**) Flow cytometric analysis of Annexin V-FITC/PI-stained 143B and SJSA-1 receiving different treatments for 24 h. The cells in Q1 to Q4 indicate dead/necrotic cells, late apoptotic/dead cells, early apoptotic cells and live cells, respectively. (**H** and **I**) Representative TUNEL staining images of 143B and SJSA-1 receiving different treatments for 24 h (**H**) and quantification of the apoptotic cell numbers (**I**). Scale bar: 50 µm. *n* = 3 *per* group. (**J**) CCK-8 analysis of the viability of 143B and SJSA-1 treated with solvent, F-AgÅPs or F-AgÅPs + Z-VAD-FMK for 24 h. *n* = 5 *per* group. (**K** and **L**) Representative images of calcein-AM/PI staining (**K**) and quantification of the percentages of live cells (**L**). Scale bar: 100 µm. *n* = 3 *per* group. (**M**) Flow cytometric analysis of Annexin V-FITC/PI-stained 143B and SJSA-1 receiving different treatments for 24 h. Data are shown as mean ± SD.^*^P < 0.05,^**^P < 0.01, ^***^P < 0.001.

Our previous study has revealed that apoptosis, but not autophagy and ferroptosis, is the major mechanism of F-AgÅPs-induced death of pancreatic and lung cancer cells 16. We thus investigated whether apoptosis mediates the killing effects of F-AgÅPs on osteosarcoma cells. Immunohistochemistry staining for TUNEL in tumor tissues from subcutaneous 143B and orthotopic SJSA-1-bearing mice showed that F-AgÅPs profoundly increased the numbers of apoptotic cells, and the effects were much higher than that of cisplatin (**Figure 5A-D**). Immunostaining for active caspase-3 (an apoptotic executive protein) also revealed that F-AgÅPs had a higher ability to induce apoptotic responses in tumor tissues from 143B-bearing mice (**Figure 5**, **E** and** F**). *In vitro*, flow cytometry after Annexin V-FITC/PI staining showed that the proportions of early apoptotic (Annexin V-FITC^+^PI^-^; Q3) and late apoptotic/dead (annexin V-FITC^+^PI^+^; Q2) 143B and SJSA-1 were markedly enhanced after exposure to IC50 doses of F-AgÅPs (**Figure 5G**). The positive effect of F-AgÅPs on apoptosis of these cells was confirmed by TUNEL staining (**Figure 5**, **H** and **I**). When the cells were co-treated with a general caspase inhibitor (Z-VAD-FMK), the F-AgÅPs-induced inhibition of survival of 143B and SJSA-1 was significantly attenuated, as revealed by CCK-8 assay (**Figure 5J**) and live/dead cell staining (**Figure 5**,** K** and **L**). The F-AgÅPs-induced increases of the percentages of early apoptotic and late apoptotic/dead cells were also profoundly reversed by this inhibitor (**Figure 5M**). Pyroptosis is a form of caspases-dependent programmed necrotic cell death 38. This involvement of pyroptosis was tested in F-AgÅPs-induced osteosarcoma cell death. The results showed that necrosulfonamide (NSA), a pyroptosis inhibitor 39, could not reverse the inhibitory effect of F-AgÅPs on survival of 143B and SJSA-1, as indicated by CCK-8 assay (**Figure S2A**) and live/dead cell staining (**Figure S2**, **A** and **B**). These results indicate that apoptosis, but not pyroptosis, mediates F-AgÅPs-induced osteosarcoma cell death.

**Figure 6:**
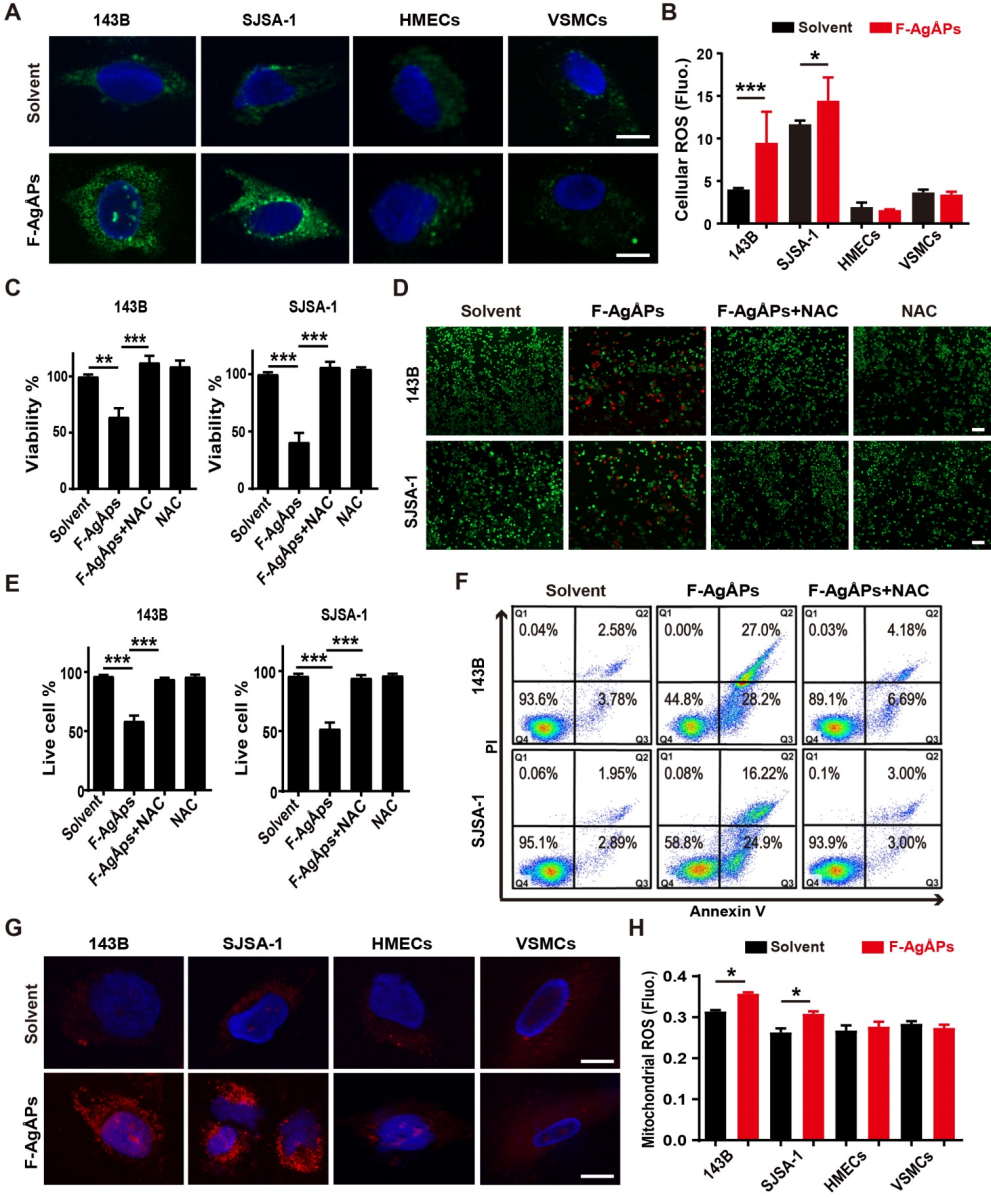
** ROS-dependent apoptosis mediates F-AgÅPs-induced osteosarcoma cell death.** (**A**) DCFH-DA staining of intracellular ROS in 143B and SJSA-1 treated with solvent or IC50 doses of F-AgÅPs for 24 h. Scale bar: 20 µm. (**B**) Intracellular ROS levels in 143B, SJSA-1, HMECs and VSMCs treated with solvent or IC50 doses of F-AgÅPs for 24 h were measured by DCFH-DA staining using a fluorescence microplate reader. Fluo: fluorescence. *n* = 5 *per* group. (**C**) CCK-8 analysis of the viability of 143B and SJSA-1 treated with solvent, F-AgÅPs, NAC or F-AgÅPs + NAC for 24 h. *n* = 5 *per* group. NAC: N-acetylcysteine. (**D** and** E**) Representative images of calcein-AM/PI double staining of 143B and SJSA-1 receiving different treatments for 24 h (**D**) and quantification of the percentages of live cells (**E**). Scale bar: 100 µm. *n* = 3 *per* group. (**F**) Flow cytometric analysis of Annexin V-FITC/PI-stained 143B and SJSA-1 receiving different treatments for 24 h. (**G**) Representative images of MitoSOX Red staining of mitochondrial ROS in 143B and SJSA-1 receiving different treatments for 24 h. Scale bar: 20 µm. (**H**) Mitochondrial ROS levels in 143B, SJSA-1, HMECs and VSMCs receiving different treatments for 24 h were tested by MitoSOX Red staining using a fluorescence microplate reader. *n* = 5 *per* group. Data are shown as mean ± SD.^*^P < 0.05,^**^P < 0.01, ^***^P < 0.001.

We then explored how F-AgÅPs induce apoptosis of osteosarcoma cells. Excess ROS production can activate apoptotic signal pathway to trigger cancer cell apoptosis 40-43. Our previous study has showed that F-AgÅPs augment ROS production in pancreatic and lung cancer cells 16. Thus, we determined whether the induction of ROS contributes to the F-AgÅPs-induced cytotoxicity against osteosarcoma cells. DCFH-DA is used as a probe to measure intracellular ROS. In presence of ROS, DCFH-DA is oxidized to green fluorescent DCF. As viewed under the fluorescence microscope, 143B and SJSA-1 showed green fluorescence after exposure to IC50 doses of F-AgÅPs for 24 h, but only very weak green signals were observed in the solvent-treated control cells (**Figure 6A**). Significant increases of fluorescent intensities in these cells after F-AgÅPs treatment were also determined by a fluorescence microplate reader (**Figure 6B**). However, IC50 doses of F-AgÅPs did not cause marked increases in ROS production in HMECs and VSMCs (**Figure 6**, **A** and** B**). These results suggest that F-AgÅPs preferentially induce ROS production in osteosarcoma cells but not in normal cells. When F-AgÅPs-treated 143B and SJSA-1 were co-incubated with an anti-oxidant agent, N-acetylcysteine (NAC), the F-AgÅPs-induced suppression of viability and promotion of apoptosis were all markedly rescued, as indicated by CCK-8 assay (**Figure 6C**), live/dead cell staining (**Figure 6**,** D** and **E**) and annexin V-FITC/PI staining with flow cytometry (**Figure 6F**). Mitochondria are the major sites of ROS production. MitoSOX Red staining indicated that F-AgÅPs induced a much higher level of mitochondrial ROS production in 143B and SJSA-1, but not in HMECs and VSMCs (**Figure 6G**). The preferential induction of mitochondrial ROS in osteosarcoma cells rather than normal cells was further confirmed by the quantitative measurement of the red fluorescence signals by a fluorescence microplate reader (**Figure 6H**). These data suggest that F-AgÅPs-induced osteosarcoma cell apoptosis is mediated by the induction of mitochondrial ROS.

Transmission electron microscopy provides details of the cellular ultrastructure. As shown in **Figure S3A**, large numbers of small particles were detected in endosomes (**i**), lysosomes (**ii**), nucleus (**iii**) and mitochondria (**iv**) of the F-AgÅPs-treated 143B cells. ICP-MS confirmed the much higher levels of silver in cell lysates and mitochondria of the F-AgÅPs-treated 143B and SJSA-1 compared with the solvent-treated control cells (**Figure S3B**). However, high contents of silver were also detected in cell lysates and mitochondria of HMECs and VSMCs after exposure to F-AgÅPs, even if 143B showed higher levels of cellular silver accumulation compared to VSMCs (**Figure S3B**). Thus, the selective promotion of mitochondrial ROS generation in osteosarcoma cells is not primarily due to their preferential uptake of F-AgÅPs compared to normal cell lines.

### F-AgÅPs alter the glycolytic phenotype and depolarize mitochondria in osteosarcoma cells, but not in healthy cells

**Figure 7:**
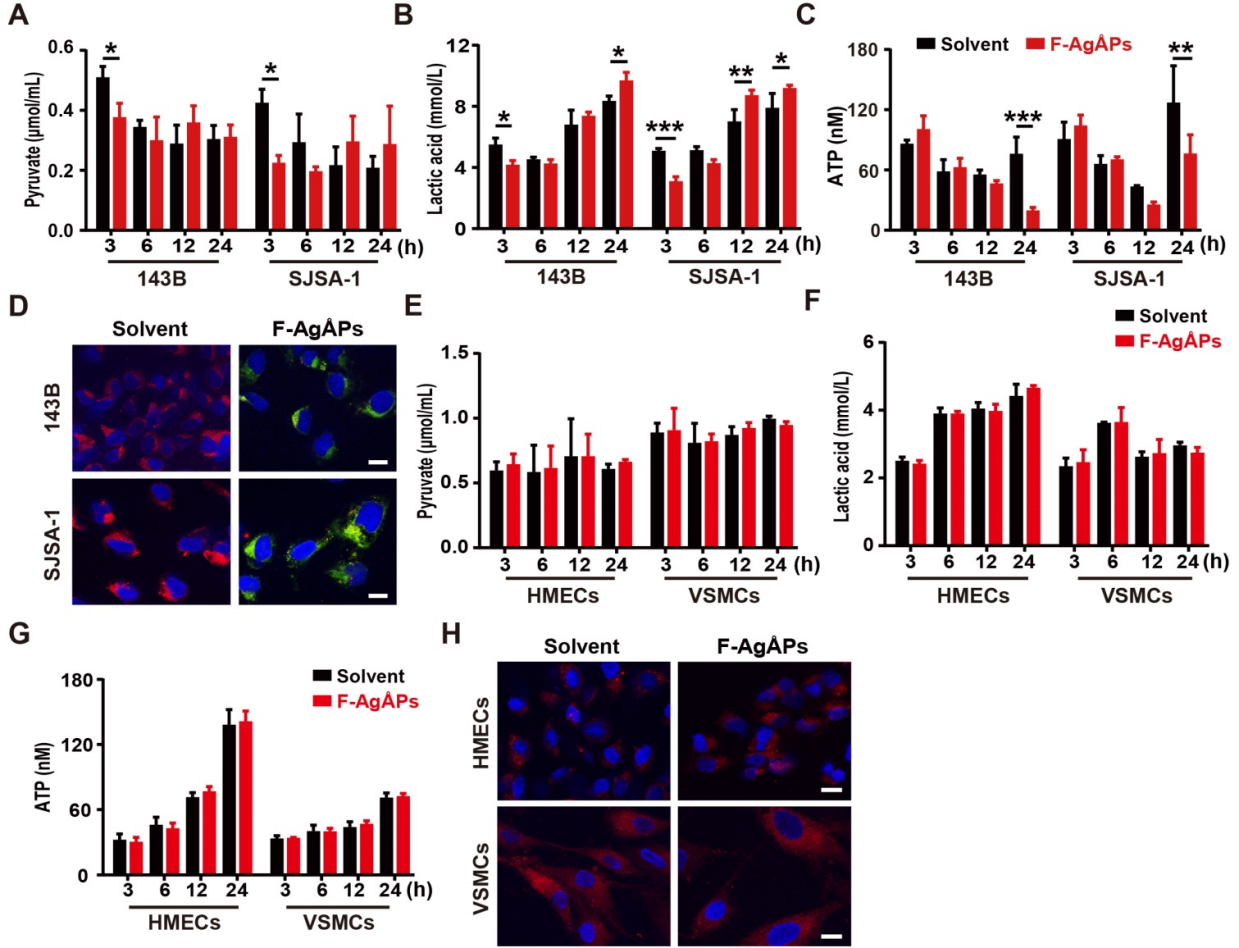
** F-AgÅPs alter the glycolytic phenotype and depolarize mitochondria in osteosarcoma cells, but not healthy cells.** (**A** and **B**) The levels of pyruvate (**A**) and lactic acid (**B**) in culture media of 143B and SJSA-1 treated with solvent or 4 ng/µL F-AgÅPs for 3 h, 6 h, 12 h or 24 h. *n* = 3 *per* group. (**C**) The levels of cellular ATP in 143B and SJSA-1 receiving different treatments for 3 h, 6 h, 12 h or 24 h. *n* = 3 *per* group. (**D**) Representative JC-1staining images of 143B and SJSA-1 receiving different treatments for 24 h. Scale bar: 50 µm. (**E-G**) The levels of pyruvate (**E**) and lactate acid (**F**) in culture media and ATP in supernatants of cell lysates (**G**) from VMSCs and HMECs treated with solvent or 4 ng/µL F-AgÅPs for 3 h, 6 h, 12 h or 24 h. *n* = 3 *per* group. (**H**) Representative JC-1staining images of VMSCs and HMECs receiving different treatments for 24 h. Scale bar: 50 µm. Data are shown as mean ± SD. ^*^P < 0.05,^ **^P < 0.01, ^***^P < 0.001.

To investigate whether the alteration of energy metabolism contributes to the selective anti-tumor efficacy of F-AgÅPs towards osteosarcoma cells, we measured glycolysis and glucose oxidation in 143B and SJSA-1 cells after exposure to 4 ng/μL F-AgÅPs for 3, 6, 12 and 24 h. The data showed that incubation of these osteosarcoma cells with F-AgÅPs for 3 h caused marked decreases in the levels of pyruvate and lactic acid in the culture media (**Figure 7**,** A** and **B**), suggesting that short-term exposure to F-AgÅPs shifts pyruvate metabolism from glycolysis and lactic acid generation towards glucose oxidation in osteosarcoma cells. The increases in ATP production after exposure to F-AgÅPs for 3 h also suggest the augmentation of glucose oxidation (**Figure 7C**). However, the levels of ATP generation in these cells dropped dramatically after 24 h of exposure to F-AgÅPs, especially in 143B, the most sensitive osteosarcoma cells to F-AgÅPs (**Figure 7C**), suggesting the possible disruption of mitochondrial function caused by F-AgÅPs. The increases of lactic acid accumulation in 143B and SJSA-1 after incubation with F-AgÅPs for 12 or/and 24 h might be a cellular response for acquiring energy to survive through increased glycolysis (**Figure 7B**). JC-1 staining showed that the solvent-treated 143B and SJSA-1 had high Δ*Ψ*_m_ (red fluorescence; **Figure 7D**). Incubation with F-AgÅPs for 24 h caused marked increases in green fluorescence (decrease in Δ*Ψ*_m_; **Figure 7D**), suggesting that F-AgÅPs induce mitochondrial dysfunction in osteosarcoma cells, consistent with the marked reduction of ATP production. F-AgÅPs at the dose of 4 ng/μL did not significantly alter the levels of production of pyruvate, lactic acid and cellular ATP in normal cell lines including HMECs and VSMCs (**Figure 7E-G**). There was also no notable change of Δ*Ψ*_m_ in these cells after exposure to F-AgÅPs (**Figure 7H**). These findings indicate that incubation of osteosarcoma cells with F-AgÅPs induces a metabolic switch from glycolysis to glucose oxidation at the early phase and finally causes mitochondrial dysfunction after long-term exposure, whereas the metabolism and mitochondrial function in normal cells are not notably disturbed by F-AgÅPs.

### Inhibition of PDK contributes to F-AgÅPs-induced ROS production, ΔΨ_m_ reduction and apoptosis of osteosarcoma cells

**Figure 8:**
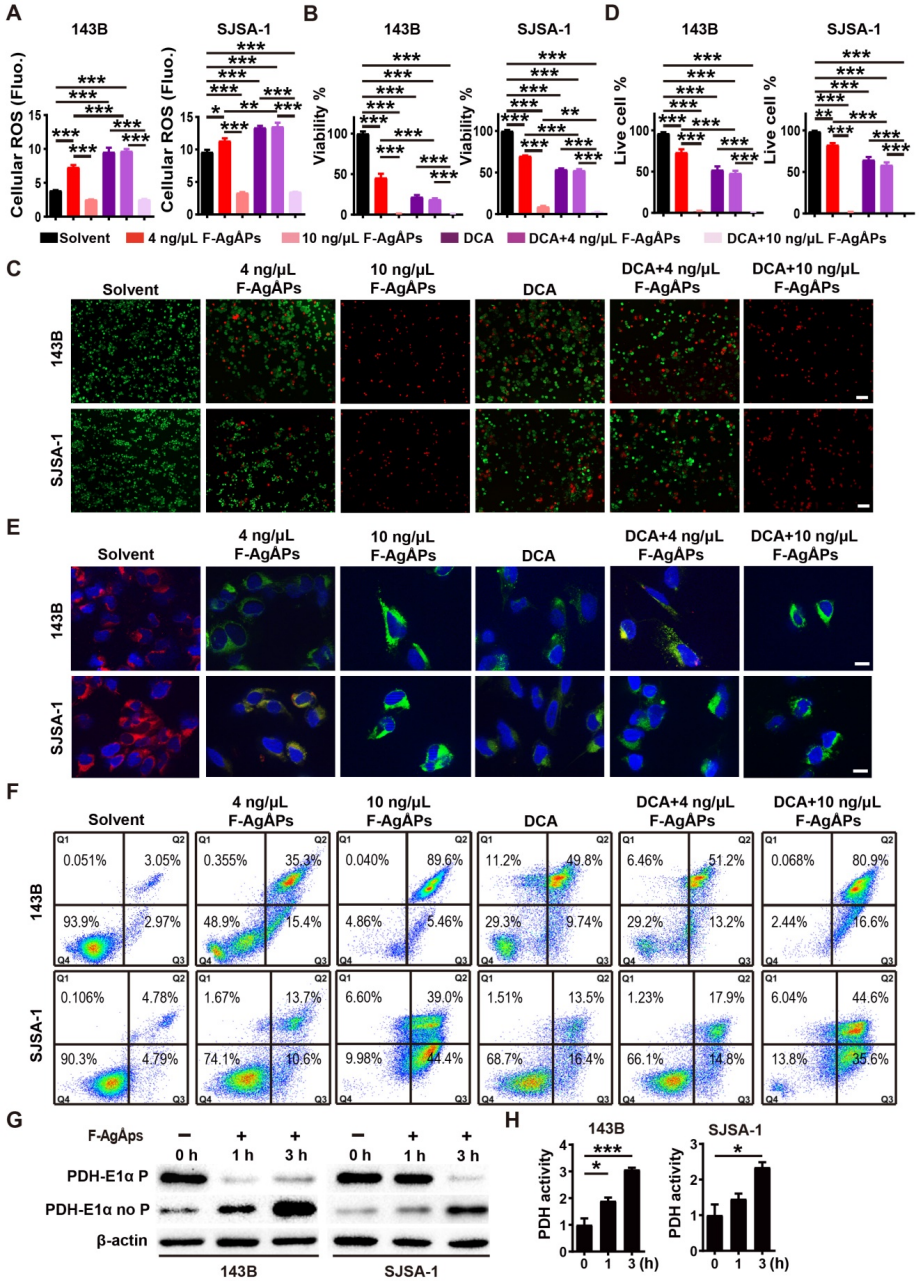
** Inhibition of PDK contributes to F-AgÅPs-induced ROS production, Δ*Ψ*_m_ reduction and apoptosis of osteosarcoma cells.** (**A**) Intracellular ROS levels in 143B and SJSA-1 receiving different treatments for 24 h were measured by DCFH-DA staining with a fluorescence microplate reader. DCA: dichloroacetate. *n* = 5 *per* group. (**B**) CCK-8 analysis of the viability of 143B and SJSA-1 receiving different treatments for 24 h. *n* = 5 *per* group. (**C** and** D**) Representative calcein-AM/PI staining images of 143B and SJSA-1 receiving different treatments for 24 h (**C**) and quantification of the percentages of live cells (**D**). *n* = 3 *per* group. (**E**) Representative JC-1 staining images of 143B and SJSA-1 receiving different treatments for 24 h. Scale bar: 50 µm. (**F**) Flow cytometric analysis of Annexin V-FITC/PI-stained 143B and SJSA-1 receiving different treatments for 24 h. (**G**) Western blotting for non-phosphorylated and phosphorylated E1α subunit of PDH protein. (**H**) PDK activity assay of 143B and SJSA-1 receiving different treatments for 1 h or 3 h.* n* = 3 *per* group. Data are shown as mean ± SD. ^*^P < 0.05,^**^P < 0.01, ^***^P < 0.001.

We next examined whether the activation of PDH (a gate-keeping mitochondrial enzyme that converts cytosolic pyruvate to mitochondrial acetyl-CoA for oxidation 25) through the inhibition of PDK is a mechanism of the F-AgÅPs-induced ROS-dependent apoptotic death of osteosarcoma cells. Dichloroacetate (DCA), a well-characterized PDK inhibitor 25, was used to treat 143B and SJSA-1. Like F-AgÅPs, DCA caused significant increases in ROS production, decreases in cell viability and Δ*Ψ*_m_, as well as increases in osteosarcoma cell apoptosis, as indicated by DCFH-DA staining assay (**Figure 8A**), CCK-8 assay (**Figure 8B**), live/dead cell staining (**Figure 8**,** C** and** D**), JC-1 staining (**Figure 8E**) and annexin V-FITC/PI staining with flow cytometry (**Figure 8F**), respectively. Co-incubation with 4 ng/μL F-AgÅPs did not induce obvious additional or overlay effects in the DCA-treated 143B and SJSA-1 cells (**Figure 8A-F**), suggesting that the inhibition of PDK is a major mechanism by which the relatively low dose of F-AgÅPs induce ROS production and cytotoxic effects in osteosarcoma cells. However, high dose (10 ng/μL) of F-AgÅPs did not induce a further increase of ROS production (**Figure 8A**), but had the ability to further decrease cell viability and Δ*Ψ*_m_, as well as augment apoptosis of the DCA-treated 143B and SJSA-1 (**Figure 8B-F**), suggesting that another mechanism independent of PDK inhibition contributes to osteosarcoma cell death after exposure to an overdose of F-AgÅPs. The very low levels of ROS were produced in cells treated with 10 ng/μL F-AgÅPs alone or with DCA + 10 ng/μL F-AgÅPs (**Figure 8A**), which were likely attributed to that most of 143B and SJSA-1 were dead after exposed to 10 ng/μL F-AgÅPs (**Figure 8F**). Western blotting showed that incubation of 143B and SJSA-1 with IC50 doses of F-AgÅPs decreased phosphorylated E1α subunit of PDH (PDH E1α) and increased the levels of non-phosphorylated PDH E1α in a time-dependent manner (**Figure 8G**), indicating that F-AgÅPs treatment causes the activation of PDH, the downstream target of PDK. The positive effect of F-AgÅPs on PDH activity was further confirmed by the PDH activity assay (**Figure 8H**). These findings indicate that the activation of PDH by inhibiting PDK contributes to F-AgÅPs-induced ROS generation, Δ*Ψ*_m_ reduction and apoptotic death of osteosarcoma cells.

## Discussion

The present study demonstrated that F-AgÅPs exhibited potent cytotoxic effects against osteosarcoma cells *in vitro*, but were well tolerated in various normal cells. *In vivo*, the administration of F-AgÅPs *via* intravenous route not only resulted in greater extents of tumor growth suppression and survival improvement in subcutaneous osteosarcoma-bearing mice, but also caused much stronger inhibitions of bone destruction and lung metastasis in orthotopic osteosarcoma-xenografted mice, as compared to the first-line chemotherapeutic drug (cisplatin) for osteosarcoma therapy. Increased tumor burden is frequently accompanied by the development of cachexia 44. Here we did not detect continued decreases in body weight and food/water consumption in the solvent-treated osteosarcoma-bearing mice with the increase of tumor burden, but these indicators were much lower in the cisplatin-treated mice, in spite of its anti-tumor effectiveness. F-AgÅPs more efficiently prevented osteosarcoma growth, without disturbing the appetite and body weight gain of mice. No any other obvious toxicities were detected in mice subjected to F-AgÅPs therapy. Our study suggests the promising prospect of F-AgÅPs as a highly efficient and safe intravenous agent for osteosarcoma therapy.

The ideal anti-cancer agent for clinical application should be capable of efficiently targeting tumor sites to fully exert their therapeutic action and meanwhile minimizing their accumulation into normal tissues, thus reducing the likelihood of toxicity 36, 45. NPs hold great superiority over small-molecule chemotherapeutic drugs partly due to their ability to accumulate more in tumor sites than healthy tissues through EPR effect 10-13. Our newly obtained F-AgÅPs (physical size: 9.38 ± 4.11 nm; hydrodynamic diameter: 15.75 ± 6.94 nm) were smaller than that our previously reported F-AgÅPs (hydrodynamic diameter: 22.8 ± 5.4 nm). The size features could enable them more efficient to kill tumor cells (smaller NPs have stronger anti-tumor effects 14-16), but maintained their ability to accumulate into tumors *via* EPR effect and avoided the rapid renal clearance (that needs sizes > 5.5 nm 8-11). F-AgÅPs were the final particles used in this study. It remains unclear how many naked AgÅPs are inside every particle of F-AgÅPs. To decipher and further optimize the coating processes, future studies are required to accurately assess the numbers of naked AgÅPs packaged in every particle of F-AgÅPs. The numbers and layers of fructose coated on each particle of F-AgÅPs are also needed further investigation.

Despite the EPR effect, high proportions of the intravenously injected NPs are still able to enter into liver and spleen, which is likely attributed to the absorption and clearance of NPs by cells of the MPS in these tissues 11, 16, 37, 45, 46. Consistent with these evidences, we found that the liver and spleen were also the major sites of F-AgÅPs accumulation in osteosarcoma-bearing mice, even if highly efficient uptake of F-AgÅPs was detected by tumors compared with various normal tissues. No notable hepatic and splenic pathological changes were detected after F-AgÅPs treatment. Nevertheless, strategies to reduce the capture of F-AgÅPs by MPS in liver and spleen may augment the tumor-targeting efficiency of F-AgÅPs. In order to avoid the potential toxicity on healthy tissues, the majority of the administrated agents are required to be eliminated efficiently from the body within a reasonable timescale 45. Herein, we found that one week after a single injection of F-AgÅPs, approximately 68% of the injected F-AgÅPs were excreted through feces. Thus, high liver uptake of F-AgÅPs may contribute to their excretion into bile and clearance from the body, thus minimizing the risks of potential long-term toxicity.

ROS are byproducts of oxygen metabolism and can be produced by mitochondrial respiratory chain, NADPH oxidases, endoplasmic reticulum, peroxisomes, etc. 47. ROS participate in various physiological processes, but are detrimental to cells when generate excessively 48. Increased ROS production by cancer cells contributes to tumor initiation and progression by inducing DNA damage, but also causes tumor inhibition by triggering senescence and apoptosis, which makes cancer cells more vulnerable to further increase of ROS compared with normal cells 49-51. Several chemotherapeutic agents such as cisplatin kill cancer cells partially owing to their ability to elevate ROS generation 47. Our study determined much stronger pro-apoptotic effects of F-AgÅPs against osteosarcoma than cisplatin and demonstrated the role of ROS as a decisive factor in F-AgÅPs-induced osteosarcoma cell apoptotic death. Interestingly, many normal cells were resistant to the efficient dose of F-AgÅPs against osteosarcoma cells and no ROS overproduction occurred in these cells after exposure to F-AgÅPs. It is worthwhile to identify the factors that cause such differences.

Compared with most normal cells, cancer cells mainly utilize aerobic glycolysis to generate ATP instead of mitochondrial oxidative phosphorylation 47, 48. The impaired mitochondrial metabolism results in mitochondrial hyperpolarization and reduces the production of mitochondria ROS, all of which contribute to the resistance of cancer cells to mitochondria-dependent apoptosis 25, 26. Chen *et al.* found that nanosilver treatment increased glycolysis in various tumor and non-tumor cells 52. In contrast to nanosilver, here we found that a short-time exposure to F-AgÅPs reduced glycolysis and promoted glucose oxidation in osteosarcoma cells, but not in normal cells, which explained why F-AgÅPs preferentially induced ROS generation and apoptosis in osteosarcoma cells, but had no obvious effects in normal cells. The switch between mitochondria-based glucose oxidative phosphorylation and cytoplasm-based glycolysis is under the control of PDH 48. The activity of PDH is controlled by PDK, which inactivates PDH and thus promotes pyruvate to be reduced to lactate (glycolysis), but not to be oxidized to acetyl-CoA (glucose oxidation) 48, 53. PDK is overexpressed in a variety of malignancies including osteosarcoma compared with nonmalignant cells 54 and inhibition of PDK with either siRNAs or the orphan drug DCA can shift the metabolism of cancer cells from glycolysis towards glucose oxidation, thus increasing mitochondrial ROS-induced apoptosis 25, 26, 48, 53. Our study suggests that F-AgÅPs represent a new agent that targets PDK to reverse the glycolytic phenotype and trigger the mitochondrial ROS-dependent apoptotic death of cancer cells.

Besides ROS-mediated mitochondria injury, silver deposition in mitochondria may also disrupt the respiratory chain and elicit excessive ROS generation to aggravate oxidative damage 55, which might be a reason why continued exposure to F-AgÅPs impaired mitochondrial energy production in osteosarcoma cells and high doses of F-AgÅPs exacerbated DCA-triggered osteosarcoma cell apoptosis. Although exposure to F-AgÅPs did not induce cytotoxicity towards normal cells/tissues at the therapeutically effective dose, future studies are needed to evaluate the long-term safety of F-AgÅPs for osteosarcoma treatment.

## Materials and Methods

All experiments in this study were approved by the Ethics Committee of Xiangya Hospital of Central South University.

### Preparation and characterization of AgÅPs and F-AgÅPs

The fabrication of AgÅPs using a self-designed, automatic, enclosed, and highly efficient evaporation-condensation system was described in detail in our recently published study 16. A modified method was developed to prepare smaller F-AgÅPs. Briefly, AgÅPs and fructose were mixed in deionized water at a concentration of 0.5 g/L and 1.0 g/L, respectively, followed by ultrasonic dispersion at 3 kW and 75 kHz for 15 s (0.5 kW and 150 kHz for 15 s in our previous study 16). After standing for 30 min, the F-AgÅPs mixture was atomized at the room temperature using an ultrasonic atomizing device at 12 kW and 15 kHz (7.5 kW, 20 kHz in our previous study 16). Then, the atomized F-AgÅPs solution was harvested in a 60 square meter condenser. AgÅPs and F-AgÅPs were photographed under a HT7700 transmission electron microscope (Hitachi, Tokyo, Japan) and their physical diameters were recorded. The hydrodynamic diameters of F-AgÅPs were measured by DLS. EDS was conducted to analyze the chemical components in AgÅPs and F-AgÅPs. A PerkinElmer Lambda750 UV-Vis-NIR spectrophotometer (Waltham, Massachusetts, USA) was applied to assess the absorbance spectra of AgÅPs and F-AgÅPs. FT-IR absorption spectra of fructose, AgÅPs and F-AgÅPs in the range of 4000-400 cm^-1^ were measured by a Nicolet iS5 spectrometer from Thermo Fisher Scientific (Madison, WI, USA).

To compare the dispersion and stability of AgÅPs and F-AgÅPs in aqueous solution, they were dissolved in deionized water and photographed until the suspensions were placed at room temperature for one month. To evaluate the dispersion and stability of F-AgÅPs in different biological media or aqueous solutions, F-AgÅPs were placed in plasma, cell culture media (including DMEM and α-MEM), normal saline, deionized water and PBS at room temperature for 15 days. The images of the F-AgÅPs solutions were obtained and silver concentrations in their supernatant were analyzed by ICP-MS.

To assess whether F-AgÅPs can release silver ions, F-AgÅPs in deionized water were left at room temperature for 15 days and then mixed with excessive HCl. AgNO_3_ solution at the same silver concentration was served as the positive control to react with HCl. The appearances of F-AgÅPs and AgNO_3_ solutions were photographed. After being left in deionized water at room temperature for 15 days and in serum at room temperature for 24 h, F-AgÅPs and AgNO_3_ preparations were centrifuged at 100, 000 × g for 2 h at 4 °C to remove silver particles or precipitates. The amounts of silver ions in the supernatant were tested by ICP-MS.

### Cell sources, culture conditions and treatments

K7M2 cell line was derived from a BALB/c mouse with lung metastatic osteosarcoma. 143B, SJSA-1, MG63, MNNG-HOS and U2OS cell lines were respectively derived from a 13-year-old girl, a 19-year-old boy, a 14-year-old boy, a 13-year-old girl and a 15-year-old girl with osteosarcoma. Two strains of primary osteosarcoma cells were respectively isolated from a 13-year-old boy and a 15-year-old boy with advanced osteosarcoma. Written informed consents were obtained from the patients and their parents. HMECs and VSMCs were derived from dermal endothelium from a newborn male donor and aorta/smooth muscle from an 11-month-old female donor, respectively. Primary osteoblasts and monocytes were isolated from new born and 6-week-old C57BL/6 mice, respectively. K7M2, U2OS, MNNG-HOS as well as primary osteosarcoma cells, monocytes and osteoblasts were cultured in high glucose DMEM (Gibco, Grand Island, USA) with 10% fetal bovine serum (FBS; Gibco). 143B was cultured in α-MEM (Hyclone, Logan, UT, USA) with 10% FBS. SJSA-1 was incubated in RPMI 1640 medium (Biological Industries, Beit Haemek, Israel) with 10% FBS. 143B and SJSA-1 were purchased from Zhong Qiao Xin Zhou Biotechnology (Shanghai, China). K7M2, MG63, MNNG-HOS and U2OS were kindly gifted from the Department of Orthopedics of Xiangya Hospital (Hunan, China). VSMCs (FuHeng Biology, Shanghai, China) and HMECs (Cell Bank of the Chinese Academy of Sciences, Shanghai, China), respectively, were incubated in F12K medium (Hyclone) with 10% FBS and in MCDB131 medium (Gibco) with 10% FBS, 10 ng/mL EGF (Peprotech, USA) and 1% GlutaMAX (Gibco). These cells were treated with solvent (control), different doses of F-AgÅPs, F-AgÅPs + Z-VAD-FMK (50 μM; MedChemExpress, Newark, USA), F-AgÅPs + NSA (5 μM; MedChemExpress), NAC (5 mM; Sigma‑Aldrich, St. Louis, MO, USA), NAC + F-AgÅPs, DCA (1.0 mM; Sigma) or F-AgÅPs + DCA for 24 h, and then subjected for the downstream assays at the indicated time points.

### CCK-8 analysis

CCK-8 assay was performed as described previously 16, 56. Briefly, cells (5 × 10^3^ cells/well) were plated and maintained in 96-well culture plates for 24 h, followed by exposure to above-described treatments for 24 h. After that, cells were then incubated for 3 h in fresh medium with CCK-8 reagent (7Sea Biotech, Shanghai, China) and the values of optical density (OD) were measured at 450 nm. The mixture of the medium and CCK-8 solution was added to four blank wells (without cells) in each plate and the OD values of these blank wells were also measured after 3 h of incubation. The viability of cells (%) = (mean OD values of treated cells - mean OD values of blank cells) / (mean OD values of control cells -mean OD values of blank cells) × 100. To compare the sensitivity of different cells to F-AgÅPs, the IC_50_ values for F-AgÅPs in these cells were calculated.

### Cell apoptosis assay

2.0 × 10^5^ cells *per* well were subjected to above-described different treatments for 24 h in 6-well culture plates. The treated cells were then stained by Annexin V-FITC/PI using a commercial kit (Yeasen Biotech, Shanghai, China) and examined by flow cytometry.

### Live/dead cell staining

The treated cells were collected, washed with assay buffer (Yeasen), incubated with 2 μM Calcein-AM (Yeasen) and 4.5 μM PI solution (Yeasen) at 37 °C for 15 min, and photographed under a Zeiss fluorescence microscope (Jena, Germany). The ratios of live (Calcein-AM^+^PI^-^) and dead (Calcein-AM^-^PI^+^) cells were calculated.

### Colony formation assay

0.2 × 10^3^ cells *per* well were treated with F-AgÅPs (2 or 4 ng/μL) or solvent for two weeks in 6-well culture plates. After that, the treated cells were stained for 5 min with 0.5% crystal violet (Solarbio, Beijing, China) and the numbers of colonies (>50 cells/colony) were counted under a Motic AE2000 inverted microscope (Xiamen, China). After taking the digital photo for each well, the colonies in each image were marked in Adobe Photoshop CS6 and the numbers of colonies were manually counted for verification.

### Measurement of ROS

After receiving different treatments, the treated cells were incubated with DCFH-DA (Beyotime, Jiangsu, China) or MitoSOX Red (Yeasen) and then analyzed using a fluorescence microscope (Zeiss) or a Varioskan LUX Multimode microplate reader (Thermo Fisher Scientific).

### JC-1 staining

To assess the changes of Δ*Ψ*_m_, the treated cells were incubated at 37 °C with JC-1 reagent (Yeasen) for 20 min and their nuclei were then stained by DAPI (Vector Laboratories, Burlingame, USA). After that, cells were imaged under a fluorescence microscope (Zeiss).

### Uptake of F-AgÅPs

After treatment with solvent or F-AgÅPs for 24 h, the treated cells were processed for transmission electron microscope observation to detect the distribution of F-AgÅPs. To assess the levels of silver within the cells and their mitochondria after F-AgÅPs treatment, the treated cells and their mitochondria were harvested and subjected to ICP-MS analysis.

### Metabolic measurements

3.0 × 10^5^ cells *per* well in a 6-well plate were treated with solvent or F-AgÅPs for 6, 12 or 24 h. The treated cells were harvested and lysed for 40 min with 1% Triton X-100. The culture media of these cells were also collected. After centrifugation (for cell lysates: 12,000 ×g for 10 min; for culture media: 400 ×g for 5 min), the intracellular ATP levels in the supernatants of cell lysates were detected using an ATP assay kit from Beyotime, and the supernatants of the culture media were subjected for the analyses of lactic acid and pyruvate using commercial kits from Jiancheng Bioengineering Institution (Nanjing, China).

### Western blotting

Western blot analysis was performed as previously described 57, 58. The antibodies targeting non-phosphorylated and phosphorylated E1α subunit of PDH were purchased from Abcam (Cambridge, Britain). Anti-beta actin and all secondary antibodies were obtained from Cell Signaling Technology (Danvers, USA).

### PDH activity

The treated cells and their culture media were collected, incubated with the reagents from a PDH activity assay kit (Sigma) and then analyzed using a microplate reader (Thermo Fisher Scientific).

### Animal studies

5-weeks-old male BALB/c nude mice were used for experiments. 5.0 × 10^6^ 143B cells in 100 μL PBS were subcutaneously injected into the mouse right flank to generate subcutaneous osteosarcoma models. For establishing orthotopic models of osteosarcoma, 5.0 × 10^5^ SJSA-1 cells in 20 μL PBS were injected into bone marrow cavity of the mouse right femur. One week after 143B or SJSA-1 injection, these mice (*n* = 6 *per* group) were injected with solvent (normal saline), F-AgÅPs (1.5 mg/kg) or cisplatin (3 mg/kg) weekly through the tail vein. The tumor width and length as well as the body weights of mice were recorded one or two times a week. For subcutaneous models, tumor volumes (V) = (length × width^2^)/2; for orthotopic models, V = 4/3π [(length + width)/4]^2^. When tumors exceeded 2,000 mm^3^, the mice were euthanized due to ethical reasons. After receiving treatments for 3 weeks, all the mice were sacrificed after blood collection by eyeball enucleation. The blood specimens (200 μL for each sample) were subjected to routine blood test. The remaining blood was centrifugated at 1,000 ×g for 15 min and the serum samples were obtained for hepatic and renal function tests. The tumor, lung, heart, brain, liver, kidney and spleen were collected, weighed, photographed and then processed for H&E staining. The tumor tissues were further subjected to immunohistochemical and TUNEL staining. The right hindlimbs of orthotopic SJSA-1-bearing mice were photographed and the femora were obtained for μCT scanning. To compare the effects of F-AgÅPs and cisplatin on long-term survival of subcutaneous 143B-bearing mice, the mice (*n* = 10 *per* group) received F-AgÅPs, cisplatin or solvent treatments as described above. Body weights were monitored twice weekly. Food and water intake were recorded twice a week from day 21 to day 28. Mice were killed when the mice were deemed too unwell or tumor burden exceeded 2,000 mm^3^.

### H&E, immunohistochemical and TUNEL staining

The paraformaldehyde-fixed, paraffin-embedded tumor and organ tissues were prepared as described previously 16. 5-μm-thick sections were made and stained with H&E (Servicebio, Wuhan, China) for histological examination. Apoptotic responses were evaluated by TUNEL staining with a commercial kit (Yeasen) and by immunohistochemical staining for active caspase-3. Immunostaining for PCNA was conducted to test cell proliferation. Images were captured with a Zeiss fluorescence microscope or an Olympus CX31 microscope (Tokyo, Japan). The numbers of positive cells or mean staining intensities for positive areas were quantified. Anti-active caspase-3, anti-PCNA and the secondary antibodies were obtained from Servicebio.

### μCT scanning

The paraformaldehyde-fixed femurs were scanned by vivaCT80 from SCANCO Medical AG (Bruettisellen, Switzerland). The resolution, current and voltage were set to 11.4 μm *per* pixel, 145 μA and 55 kV, respectively. The images of the whole femurs were reconstructed by NRecon and visualized by µCTVol v2.2.

### Pharmacokinetics, distribution and excretion of F-AgÅPs

Male orthotopic SJSA-1 xenografts-bearing mice were randomly divided into ten groups (*n* = 5 *per* group). Five mice were killed immediately after collection of blood by eyeball enucleation. F-AgÅPs (1.5 mg/kg) were injected into the tail vein of the remaining mice. After collection of blood at 5 min, 15 min, 0.5 h, 1 h, 6 h, 12 h, 1 d, 3 d and 7 d after F-AgÅPs injection, the mice were killed. After centrifugation for 15 min at 1,000 × g, serum was harvested from blood and then subjected to ICP-MS. The tumor, lung, heart, brain, liver, kidney, spleen, femur, were collected, weighed and prepared for ICP-MS. 24 h-urine and -feces were collected from five mice injected with F-AgÅPs for 1 to 7 days. After being weighed, these samples were also processed for ICP-MS.

### Statistical analysis

Data were analyzed with GraphPad Prism 8 and presented as mean ± SD. Unpaired, two tailed Student's *t*-test and one- or two-way analysis of variance (ANOVA) followed by Bonferroni *post hoc* test were used for two-group and multiple-group comparisons, respectively. Survival curves generated by the Kaplan-Meier method were compared by the Log-rank (Mantel-Cox) test. *P* < 0.05 was considered statistically significant.

## Supplementary Material

Supplementary figures and tables.

## Abbreviations

ROS: reactive oxygen species
PDK: pyruvate dehydrogenase kinase
AgÅPs: Ångstrom-scale silver particles
F-AgÅPs: fructose-coated AgÅPs
NPs: nanoparticles
EPR: enhanced permeability and retention
Δ*Ψ*_m_: mitochondrial membrane potential
PDH: pyruvate dehydrogenase
DLS: dynamic light scattering
EDS: energy dispersive spectroscopy
UV-Vis-NIR: ultraviolet-visible near-infrared
LSPR: localized surface plasmon resonance
FT-IR: fourier-transform infrared
PBS: phosphate buffer saline
ICP-MS: inductively coupled plasma mass spectrometry
CCK-8: cell counting kit-8
IC50: half-maximal inhibitory concentration
HMECs: human microvascular endothelial cells
VSMCs: vascular smooth muscle cells
PI: propidium iodide
H&E: hematoxylin and eosin
PCNA: proliferating cell nuclear antigen
μCT: microcomputed tomography
RBC: red blood cells
PLT: platelets
HGB: hemoglobin
CRE: creatinine
BUN: blood urea nitrogen
MPS: mononuclear phagocyte system
NSA: necrosulfonamide
NAC: N-acetylcysteine
DCA: dichloroacetate
siRNA: small interfering ribonucleic acid
FBS: fetal bovine serum
EGF: epidermal growth factor
OD: optical density
ANOVA: analysis of variance
