# Supplementary material for: Fructose-coated Angstrom silver inhibits osteosarcoma growth and metastasis via promoting ROS-dependent apoptosis through the alteration of glucose metabolism by inhibiting PDK
Source: Theranostics. 2020 Jun 19;10(17):7710–29. doi: 10.7150/thno.45858 (PMC7359101; doi:10.7150/thno.45858)
Supplement: Supplementary file 1 — Supplementary figures and tables. [file thnov10p7710s1.pdf]

Supporting Information

Fructose-coated Ångstrom silver inhibits osteosarcoma growth and metastasis via promoting ROS-dependent apoptosis through the alteration of glucose metabolism by inhibiting PDK

Xiong-Ke Hu, Shan-Shan Rao, Yi-Juan Tan, Hao Yin, Ming-Jie Luo, Zhen-Xing Wang, Jin-Hua Zhou, Chun-Gu Hong, Zhong-Wei Luo, Wei Du, Ben Wu, Zi-Qi Yan, Ze-Hui He, Zheng-Zhao Liu, Jia Cao, Yang Wang, Wei-Yi Situ, Hao-Ming Liu, Jie Huang, Yi-Yi Wang, Kun Xia, Yu-Xuan Qian, Yan Zhang, Tao Yue, Yi-Wei Liu, Hong-Qi Zhang, Si-Yuan Tang, Chun-Yuan Chen, Hui Xie**

***** Corresponding authors: Hui Xie (huixie@csu.edu.cn); Chun-Yuan Chen (chency19@csu.edu.cn).

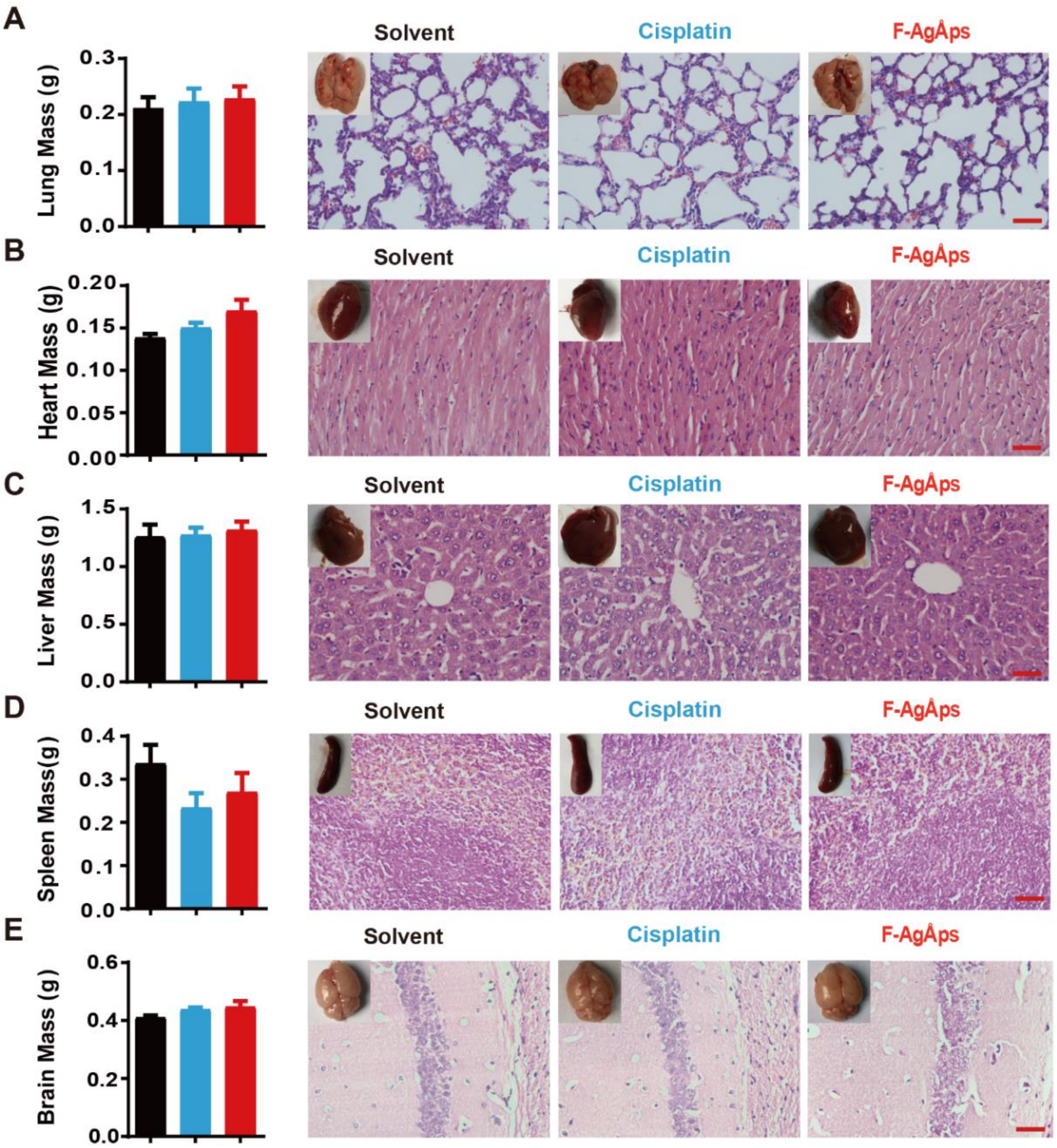

Figure S1. No obvious toxicities are induced in osteosarcoma-bearing mice after intravenous injection of F-AgÅPs. (**A-E**) Weights, gross observation and H&E staining images of lung (**A**), heart (**B**), liver (**C**), spleen (**D**) and brain (**E**) in subcutaneous 143B xenograft-bearing mice treated with solvent, F-AgÅPs or cisplatin for 21 days. Scale bar: 100 μm. $n = 6$ *per* group. Scale bar: 100 μm. Data are shown as mean ± SD. $^{*}P < 0.05$, $^{**}P < 0.01$, $^{***}P < 0.001$.

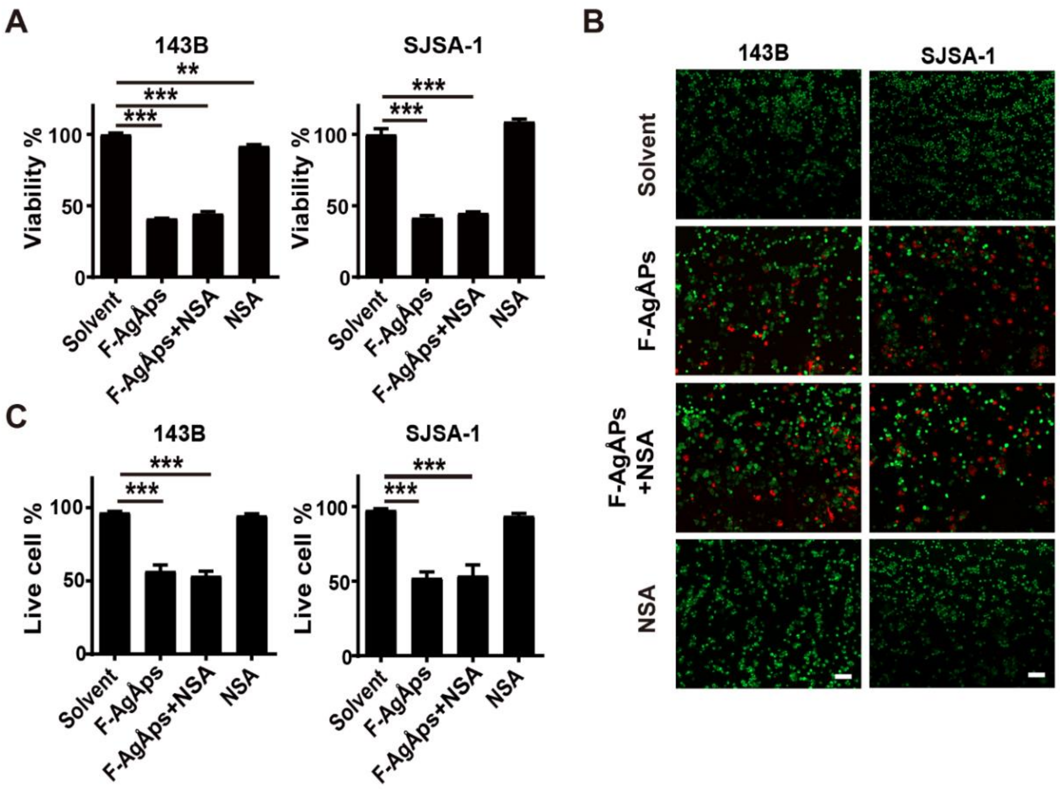

Figure S2. Pyroptosis is not involved in F-AgÅPs-induced osteosarcoma cell death. (**A**) CCK-8 analysis of the viability of 143B and SJSA-1 treated with solvent, F-AgÅPs, NSA or F-AgÅPs + NSA for 24 h. NSA: necrosulfonamide. n = 5 *per* group. (**B**) Representative images of calcein-AM/PI staining of 143B and SJSA-1 receiving different treatments for 24 h. Scale bar: 100 μm. (**C**) Quantification of the percentages of live cells in (**B**). n = 3 *per* group. Data are shown as mean ± SD. $^{*}P < 0.05$, $^{**}P < 0.01$, $^{***}P < 0.001$.

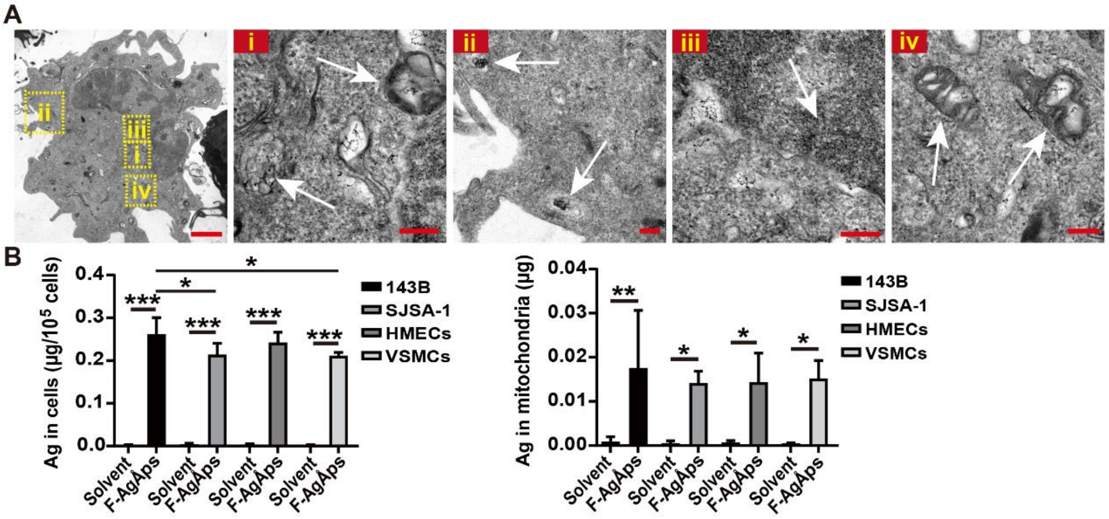

Figure S3. Internalization of F-AgÅPs by osteosarcoma cells and healthy cells. (**A**) Representative transmission electron microscopy images of 143B treated with F-AgÅPs for 24 h. White arrows indicate the accumulation of F-AgÅPs in cellular endosomes (**i**), lysosomes (**ii**), nucleus (**iii**) and mitochondria (**iv**). Scale bar: 2 µm. (**B**) Silver levels in cell lysates and mitochondria of 143B, SJSA-1, VMSCs and HMECs treated with solvent or F-AgÅPs for 24 h were measured by ICP-MS. $n = 4$ *per* group. Data are shown as mean ± SD. $^{*}P < 0.05$, $^{**}P < 0.01$, $^{***}P < 0.001$.

Table S1 Hematologic indexes in mice treated with solvent, cisplatin and F-AgÅPs.

Hematologic indexes	Solvent	Cisplatin	F-AgÅPs
Red blood cells (RBC) 10^{12}/L	7.19 ± 0.33	6.21 ± 1.67	7.48 ± 0.23
Hemoglobin (HGB) g/L	113.80 ± 1.79	96.00 ± 25.85	116.00 ± 3.61
Hematokrit (HCT) %	35.56 ± 0.93	29.18 ± 7.84	35.42 ± 1.03
White blood cells (WBC) 10^9/L	12.96 ± 4.85	14.14 ± 1.99	12.28 ± 4.41
Platelets (PLT) 10^9/L	560.00 ± 96.09	515.60 ± 164.30	631.00 ± 56.45
Neutrophil (NEUT%) %	8.28 ± 2.72	14.76 ± 5.72	16.34 ± 8.78
Lymphocytes (LYMPH%) %	83.70 ± 2.08	80.28 ± 4.70	76.62 ± 8.72
Monocyte (MONO%) %	6.72 ± 3.30	4.18 ± 2.39	5.48 ± 3.47

n = 5 *per* group. Data are shown as mean ± SD.
